# Linking Gut Microbiota, Mitochondrial Redox Dysfunction, and Ferroptosis in Cardiometabolic Diseases: A Narrative Review of Mechanistic Evidence and Redox-Targeted Interventions

**DOI:** 10.3390/antiox15070803

**Published:** 2026-06-27

**Authors:** Yirui Chen, Jingzhi Zhu, Hongxin Gui, Mingyuan Liu, Ye Zhang, Zimu Wu, Chang Liu, Mengyang Wang

**Affiliations:** 1 School of Public Health and Health Sciences, Tianjin University of Traditional Chinese Medicine, Tianjin 301617, China; ciandata@163.com (Y.C.); ghostcixi@163.com (H.G.);; 2 Clinical Medical School of Acupuncture, Moxibustion and Rehabilitation, Guangzhou University of Chinese Medicine, Guangzhou 510006, China; 3 Centre for Epidemiology and Biostatistics, Melbourne School of Population and Global Health, University of Melbourne, Melbourne, VIC 3010, Australia; y.zhang@unimelb.edu.au; 4 School of Public Health and Preventive Medicine, Monash University, 553 St Kilda Road, Melbourne, VIC 3004, Australia; zimu.wu1@monash.edu; 5 School of Sport Science, Beijing Sport University, Beijing 100084, China

**Keywords:** redox modulation, gut microbiota, microbial metabolites, mitochondrial dysfunction, ferroptosis, lipid peroxidation, cardiometabolic diseases, natural antioxidants, probiotics

## Abstract

Cardiometabolic diseases are increasingly understood as disorders involving compartment-specific redox disruption rather than a uniform excess of reactive oxygen species. This narrative review synthesizes evidence for a proposed gut microbiota–mitochondria ferroptosis framework in which dysbiosis-derived lipopolysaccharide, trimethylamine N-oxide, short-chain fatty acids, bile acids, and tryptophan metabolites may modulate mitochondrial reactive species production, antioxidant defenses, iron handling, lipid peroxide detoxification, and inflammatory signaling. The reference set was assembled through searches of PubMed and Web of Science Core Collection, supplemented by targeted Google Scholar searches and citation chaining during manuscript preparation and revision through June 2026 and was organized around microbial metabolites, mitochondrial redox biology, ferroptosis pathways, disease-specific evidence, and redox-targeted interventions. Because this is a narrative synthesis rather than a systematic review, the framework should be interpreted as hypothesis-generating rather than as a systematically validated pathological model. Across atherosclerosis, diabetic cardiomyopathy, metabolic dysfunction-associated steatotic liver disease, obesity-associated insulin resistance, chronic kidney disease, and cardiorenal metabolic injury, the most consistent mechanistic links involve mtROS, impaired mitophagy, glutathione/GPX4 and SLC7A11 dysfunction, ACSL4-dependent lipid peroxidation, Nrf2 signaling, NLRP3 activation, and cGAS-STING-associated inflammation, although human causal evidence remains uneven. Importantly, much of the current literature supports local links within this sequence rather than a fully verified dysbiosis–metabolite–mitochondria ferroptosis–organ dysfunction chain in the same study. We therefore emphasize evidence tiers, terminology discipline, and biomarker requirements when interpreting ferroptosis-sensitive injury. Polyphenols, flavonoids, probiotics, postbiotics, melatonin, CoQ10-related strategies, mitochondria-targeted antioxidants, and ferroptosis-sensitive approaches may be most translatable when paired with microbiome, metabolomic, lipidomic, pharmacokinetic, and redox biomarkers.

## 1. Introduction

Cardiometabolic diseases comprise a family of disorders in which obesity, insulin resistance, dyslipidemia, hypertension, atherosclerosis, metabolic dysfunction-associated steatotic liver disease (MASLD, formerly referred to as NAFLD in much of the earlier literature), diabetic cardiomyopathy, and chronic kidney disease reinforce one another through shared metabolic and inflammatory circuits. Contemporary reviews of hypertension, diabetes, chronic kidney disease, MASLD, and cardiovascular disease consistently identify oxidative stress and inflammation as cross-disease mechanisms, but the field has moved from the older concept of indiscriminate reactive oxygen species (ROS) excess toward a more granular view of redox biology [1,2,3,4,5]. ROS, reactive nitrogen species, reactive sulfur species, lipid radicals, and oxidized phospholipids are now interpreted as active species whose biological effects depend on site of production, duration, buffering capacity, lipid context, iron availability, and crosstalk with regulated cell death. This shift is especially important for cardiometabolic disease, where a modest mitochondrial signal may support adaptation, whereas persistent mitochondrial ROS (mtROS), lipid peroxide propagation, and iron-catalyzed chemistry can contribute to irreversible tissue injury.

The gut microbiota has become a plausible upstream organizer of this redox landscape. Dysbiosis, impaired intestinal barrier integrity, and altered microbial metabolism can expose host tissues to lipopolysaccharide (LPS), trimethylamine N-oxide (TMAO), modified bile acids, short-chain fatty acids (SCFAs), indoles, phenolic metabolites, and other small molecules. Reviews focused on the gut–liver axis and MASLD emphasize that microbial signals can regulate hepatic oxidative stress, lipid peroxidation, bile acid signaling, and ferroptosis [6,7,8,9]. In parallel, ferroptosis, an iron-dependent form of regulated cell death characterized by phospholipid peroxidation, has emerged as a redox-sensitive injury process relevant to metabolic organ injury, including MASLD, diabetic nephropathy, endothelial dysfunction, and myocardial injury [10,11,12,13,14]. The convergence of gut-derived metabolites, mitochondrial redox imbalance, lipid peroxidation, and ferroptosis therefore provides a sharper mechanistic frame than the broad phrase “oxidative stress” alone.

The attraction of a staged framework is that it helps explain why single-node interventions often underperform. A patient with obesity and insulin resistance may simultaneously show a high-fat-diet-shaped microbiome, reduced epithelial resilience, altered bile acid pools, hepatic mitochondrial overload, endothelial nitric oxide loss, renal oxidative injury, and systemic inflammatory priming. Treating only one downstream marker, such as total ROS, does not necessarily normalize the ecological and metabolic inputs that continue to feed tissue injury. Conversely, improving microbial metabolite balance may be insufficient if target tissues have already developed mitochondrial quality control failure, labile iron accumulation, or defective lipid peroxide repair. The proposed gut microbiota–mitochondria ferroptosis framework therefore emphasizes sequence and hierarchy: upstream dysbiosis and barrier disruption may provide triggers, mitochondria may set the redox gain, ferroptosis-sensitive pathways may influence whether lipid stress becomes injurious, and inflammation may influence whether damage resolves or becomes chronic [2,6,10,15].

This framework also helps position redox-targeted interventions in a more contemporary way. Many compounds labeled as antioxidants are pleiotropic regulators of microbial communities, host xenobiotic metabolism, Nrf2-dependent cytoprotection, mitochondrial biogenesis, mitophagy, lipid remodeling, iron handling, and immune polarization. This is evident in reviews of flavonoids, phytochemicals, and traditional-medicine-derived cardiometabolic interventions [16,17,18,19]. Intervention studies involving ginsenosides, polysaccharides, curcumin, and probiotics further show that antioxidant effects often overlap with microbiota, mitochondrial, and ferroptosis-related regulation [20,21,22,23]. Their therapeutic relevance depends less on test tube radical scavenging capacity than on whether they have plausible mechanisms of action, acceptable pharmacokinetics, microbial or host bioactivation, tissue access, and the ability to preserve endogenous redox defenses without suppressing adaptive ROS signaling. For this reason, antioxidant candidates should be discussed together with microbiota-targeted, mitochondria-targeted, and ferroptosis-sensitive strategies rather than as a separate nutritional appendix.

**Figure 1:**
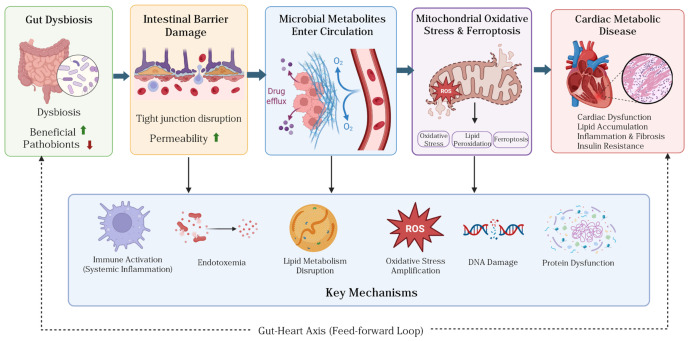
Graphical abstract of the proposed gut microbiota–mitochondria ferroptosis framework in cardiometabolic diseases. Gut dysbiosis and barrier disruption may alter microbial products and metabolites, including LPS, TMAO, SCFAs, bile acids, and tryptophan-derived metabolites, which can reach cardiometabolic target tissues and reshape mitochondrial redox homeostasis, lipid peroxidation, ferroptosis sensitivity, and inflammation. Redox-targeted interventions may engage microbial, mitochondrial, lipid peroxide, ferroptosis-sensitive, or inflammatory checkpoints, but the figure is a conceptual synthesis rather than proof of a complete causal chain. Created in BioRender. Mingyuan, L. (2026) https://BioRender.com/00dssst, accessed on 24 May 2026.

This narrative review synthesizes evidence around the gut microbiota–mitochondria ferroptosis framework in cardiometabolic diseases and evaluates redox-targeted interventions through that framework. The central argument is that gut dysbiosis may represent an upstream source of metabolic and inflammatory pressure; mitochondria may translate these cues into spatially organized redox stress; ferroptosis-sensitive lipid peroxidation and inflammation may contribute to tissue injury; and interventions are most promising when they restore microbial ecology, mitochondrial quality control, lipid peroxide detoxification, and inflammatory resolution rather than simply scavenging ROS. Figure 1 summarizes the proposed framework and identifies the main microbial metabolites and tissue checkpoints discussed throughout the review.

The review makes three specific contributions. First, it connects microbial metabolites directly to mitochondrial redox imbalance and ferroptosis rather than treating the gut microbiota as a separate upstream risk factor. Second, it compares how the same framework appears across vascular, cardiac, hepatic, adipose, and renal metabolic injury, thereby distinguishing shared mechanisms from tissue-specific manifestations. Third, it reframes natural antioxidants, dietary bioactives, probiotics, postbiotics, and mitochondria-targeted compounds as checkpoint-modifying redox interventions that should be evaluated by mechanism, metabolite exposure, delivery, biomarkers, and disease-stage specificity. This organization is intended to support testable hypotheses for biomarker-driven and microbiota-informed cardiometabolic interventions.

The framework proposed here builds on, rather than replaces, established gut–organ models. The gut–liver axis in MASLD describes how portal delivery of microbial ligands, bile acids, ethanol-related products, and inflammatory signals may shape hepatic steatosis, oxidative stress, and fibrosis [6,7]. The gut–kidney axis emphasizes uremic dysbiosis, microbial toxin accumulation, intestinal barrier injury, and systemic inflammation in chronic kidney disease and diabetic kidney disease [2,24,25]. Separately, mitochondria ferroptosis models describe how mitochondrial dysfunction, lipid peroxide propagation, iron handling, GPX4 insufficiency, and inflammatory cell death may contribute to metabolic organ injury [10,13,15,26].

The added value of the present synthesis is to connect these partially overlapping models into a redox sequence that can be tested experimentally. In this view, gut-derived exposures are candidate upstream inputs that may alter mitochondrial redox tone and lipid peroxide vulnerability. Mitochondria may act as signal amplifiers that influence whether microbial and metabolic cues remain adaptive or become damaging. Ferroptosis is not treated only as a terminal cell death label; it is interpreted as the point at which lipid peroxidation, iron chemistry, antioxidant failure, and inflammation may become self-reinforcing. This sequence remains incompletely proven as an intact human disease pathway. Redox-targeted interventions are therefore evaluated according to whether they engage one or more checkpoints in this proposed sequence, not according to generic radical-scavenging capacity alone [27,28,29,30].

## 2. Scope and Narrative Literature Approach

This review was designed as a mechanism-centered narrative synthesis rather than a systematic review. The literature search was conducted across PubMed and Web of Science Core Collection, with Google Scholar used as a supplementary source for citation discovery and difficult-to-index literature. The initial searches were conducted on May 2026, and all sources were updated through June 2026. Focused searches covered gut microbiota or microbial metabolites, mitochondrial dysfunction, oxidative stress, redox homeostasis, ferroptosis, lipid peroxidation, cardiometabolic diseases, and redox-targeted interventions. Representative search concepts included “gut microbiota”, “microbial metabolites”, “TMAO”, “short-chain fatty acids”, “bile acids”, “tryptophan metabolites”, “mitochondrial dysfunction”, “mitochondrial ROS”, “oxidative stress”, “ferroptosis”, “GPX4”, “SLC7A11”, “ACSL4”, “lipid peroxidation”, “cardiometabolic disease”, “atherosclerosis”, “diabetes”, “diabetic cardiomyopathy”, “MASLD”, older fatty liver terminology such as “NAFLD” and “MAFLD”, “chronic kidney disease”, “antioxidants”, “polyphenols”, “probiotics”, and “postbiotics”. Searches were not restricted by study design because the review aimed to integrate mechanistic, translational, and clinical evidence. The full search strings and screening summary are provided in Supplementary Table S3.

The searches identified a total of 1273 records across all sources. After removal of duplicates, 696 unique records remained for screening. Of these, 150 candidate articles underwent closer evaluation, and 112 unique references were included in the final manuscript; the remaining candidate records were not cited because they were less directly related to cardiometabolic disease, duplicated mechanisms already covered by more directly relevant sources, focused on unrelated organ systems without transferable redox mechanisms, lacked a microbiota, mitochondrial, ferroptosis, or intervention component, or provided review-level statements superseded by primary studies or more recent syntheses. Additional references were identified by citation chaining from highly relevant reviews, mechanistic studies, randomized trials, Mendelian randomization studies, and meta-analyses. Screening and literature curation were performed by Y.C., J.Z. and H.G.; disagreements about inclusion or interpretation were resolved by discussion with M.W., with K.M. and M.L. contributing to evidence table checking. English-language full-text publications were used for the cited evidence set.

Priority was given to studies and reviews relevant to cardiovascular, metabolic, hepatic, renal, and diabetic disease contexts, while basic mechanistic articles were retained when they clarified redox-active species, antioxidant mechanisms of action, mitochondrial redox regulation, GPX4/SLC7A11 biology, Nrf2 signaling, iron metabolism, mtDNA release, NLRP3 activation, or microbial metabolite signaling. The final cited set includes experimental studies, translational reports, clinical association studies, randomized trial evidence where available, Mendelian randomization evidence for selected metabolites, and contemporary reviews covering gut microbiota, MASLD, diabetes, vascular dysfunction, chronic kidney disease, natural antioxidants, probiotics, and ferroptosis-related cell death. The main tables are organized as mechanism-centered syntheses: microbial metabolites (Table 2), molecular pathways (Table 3), disease-specific evidence (Table 4), intervention strategies (Table 5), and translational challenges (Table 6).

Although multiple bibliographic sources were searched, this narrative review did not employ a pre-registered protocol, exhaustive systematic database coverage, duplicate independent screening, formal PRISMA procedures, or structured risk-of-bias assessment. Because the review was not based on a pre-registered systematic search strategy, selection bias cannot be excluded. Conflicts in the literature were not resolved by a formal voting procedure; instead, human longitudinal, genetic, interventional, multi-omics, and causal-manipulation studies were weighted more heavily than cross-sectional associations or single-marker cell studies when interpreting strength of evidence. The proposed gut microbiota–mitochondria ferroptosis framework should therefore be read as an integrative and hypothesis-generating model, not as a systematically validated pathological pathway. This limitation is important because much of the mechanistic evidence derives from cell systems, high-fat-diet- or toxin-induced animal models, genetic models, and short-term interventions that may not fully reproduce human diet, medication exposure, comorbidity, microbiome heterogeneity, disease duration, sex differences, or tissue-specific pharmacokinetics. Because the review was not based on a pre-registered systematic search strategy, selection bias cannot be excluded. The cited literature should therefore be interpreted as a mechanism-centered evidence map rather than an exhaustive or quantitatively graded assessment of the field.

Because the topic cuts across several fields, we organized the evidence around mechanistic modules rather than disease silos: (i) gut dysbiosis and microbial metabolites; (ii) mitochondrial redox dysfunction and quality control; (iii) ferroptosis and lipid peroxide detoxification; (iv) inflammatory amplification; (v) disease-specific manifestations; and (vi) redox-targeted interventions. Particular attention was given to natural and synthetic antioxidants, dietary bioactives, antioxidant metabolism, pharmacodynamic and pharmacokinetic constraints, and compartment-specific redox modulation. Human association studies were used mainly to identify clinically plausible links; animal models were used to discuss mechanistic directionality; cell models were used to define intracellular redox and ferroptosis pathways; and intervention studies were used to evaluate therapeutic nodes. Findings from cellular and animal studies were interpreted cautiously and were not assumed to translate directly to humans unless supported by convergent clinical, biomarker, genetic, or intervention evidence.

Evidence was considered stronger when microbial changes were paired with metabolomic or lipidomic readouts, when mitochondrial phenotypes were measured beyond generic ROS assays, and when ferroptosis was supported by multiple markers rather than by a single change in GPX4 or malondialdehyde. Evidence was considered weaker when it relied only on taxonomic microbiome shifts, total ROS assays, isolated changes in one antioxidant protein, or disease models with limited human analogy. This layered approach is important because the same molecular term can mean different things across disciplines. For example, “oxidative stress” may refer to total ROS, mitochondrial superoxide, lipid peroxidation, oxidized glutathione, protein carbonylation, or redox-sensitive transcriptional remodeling. Similarly, “gut microbiota regulation” may describe taxonomic shifts, metabolite changes, barrier effects, or functional pathway alterations. Throughout the review, we therefore emphasize mechanistic interpretation rather than keyword co-occurrence alone.

We also distinguished local-chain evidence from complete-framework evidence (Table 1). This distinction is central to the interpretation of the review. Evidence that a microbial metabolite is associated with disease, that mitochondria are dysfunctional in the same disease, and that ferroptosis markers are altered in another model does not by itself prove an intact dysbiosis–metabolite–mitochondria ferroptosis–organ dysfunction pathway. Throughout the disease-specific sections, “ferroptosis” is reserved for studies with convergent evidence such as lipid peroxide detection, labile iron or iron-handling readouts, GPX4/SLC7A11/ACSL4 or ferritinophagy markers, rescue by ferroptosis inhibitors or genetic manipulation, and exclusion of competing death pathways where feasible. When evidence is based on multiple markers without rescue, we use “ferroptosis-associated injury”; when evidence is limited to lipid peroxidation or single-marker changes, we use “ferroptosis-sensitive” or “ferroptosis-like phenotype”.

## 3. Gut Microbiota as an Upstream Regulator of Redox Homeostasis

### 3.1. Dysbiosis, Barrier Dysfunction, and Metabolic Endotoxemia

The intestinal barrier influences whether microbial products remain luminal signals or become systemic inflammatory triggers. High-fat diets, hyperglycemia, uremic toxins, alcohol exposure, environmental toxicants, aging, and sedentary behavior can disrupt tight junctions, alter mucus production, reduce beneficial microbial communities, and promote translocation of LPS and other pathogen-associated molecular patterns. In MASLD, TLR4-related mechanisms have been discussed as a therapeutic target, and LPS-related experimental models show how inflammatory stimuli can connect liver, kidney, and myocardial injury to ferroptosis-sensitive redox pathways [31,32,33,34]. Environmental and metabolic stress studies further support the idea that barrier injury, microbiome disruption, oxidative stress, and lipid remodeling frequently occur together rather than as isolated abnormalities [35,36,37,38]. In this setting, redox imbalance is not merely a by-product of inflammation; it is part of the signaling architecture that links gut barrier failure to mitochondrial stress in peripheral tissues.

Metabolic endotoxemia is particularly relevant to cardiometabolic disease because it is associated with low-grade chronic inflammation rather than an acute septic phenotype. In endothelial cells and macrophages, LPS can increase NADPH oxidase activity and mtROS, reduce nitric oxide bioavailability, promote adhesion molecule expression, and facilitate foam cell formation. In hepatocytes, Kupffer cells, and stellate cells, LPS/TLR4 signaling has been linked to steatosis, inflammation, fibrogenesis, and ferroptosis-sensitive lipid peroxide accumulation. In renal tubular cells and cardiomyocytes, LPS-related signaling can converge on mitochondrial quality control, Nrf2 activity, SLC7A11/GPX4 expression, and inflammasome activation [39,40,41,42]. These observations support the view that intestinal permeability is a redox-relevant cardiometabolic risk marker and candidate intervention point rather than a gastrointestinal side issue.

Barrier dysfunction also changes the pharmacology of redox-targeted interventions. Many polyphenols, polysaccharides, and probiotic-derived products first act at the mucosal surface, where they can influence mucus thickness, secretory IgA, epithelial tight-junction proteins, antimicrobial peptide expression, and local macrophage or dendritic cell tone. If this first checkpoint is improved, systemic oxidative burden may decrease before any direct antioxidant action occurs in the heart, liver, or kidney. Conversely, a disrupted barrier can transform otherwise beneficial microbial communities into sources of inflammatory ligands by allowing excess translocation. This duality explains why microbiota-targeted therapies may show variable effects across models: their impact depends on epithelial integrity, dietary substrate availability, bile acid composition, medication exposure, and the pre-existing inflammatory state [24,43,44,45,46].

### 3.2. TMAO, SCFAs, Bile Acids, and Tryptophan-Derived Metabolites

Microbial metabolites differ in direction and context of effect. TMAO, generated through microbial trimethylamine production and hepatic flavin monooxygenase activity, has been associated with atherosclerosis, macrophage lipid accumulation, platelet reactivity, endothelial dysfunction, renal injury, stroke risk, and metabolic inflammation. In macrophage foam cells, TMAO has been reported to promote oxidative stress and lipid accumulation through Nrf2/ABCA1-related signaling, while pediatric evidence links TMAO to iron-related renal tubular dysfunction in a disease context characterized by iron burden [47,48]. Mechanistically, TMAO has been proposed to influence oxidative stress, cholesterol efflux, bile acid metabolism, and mitochondrial function. However, human interpretation is controversial. Bidirectional Mendelian randomization did not support a robust causal effect of genetically predicted TMAO on several cardiometabolic outcomes after multiple-testing correction, and population-level data indicate that diet, renal function, host metabolism, and microbiome composition all influence circulating TMAO and its precursors [49,50,51,52,53]. TMAO is therefore best treated as a context-dependent microbial-host co-metabolic signal and possible disease modifier rather than as a single universal toxin.

SCFAs such as acetate, propionate, and butyrate generally support epithelial integrity, mucus production, regulatory immune tone, and metabolic homeostasis through G-protein-coupled receptors, histone deacetylase inhibition, AMPK activation, and mitochondrial substrate provision. For example, acetate supplementation has been investigated in the setting of diesel-exhaust-induced gut microbiome dysbiosis, and fermentation products from marine glycosaminoglycans altered antioxidant status and gut microbiota in high-fat-diet-fed mice [35,54]. But SCFAs are not uniformly protective in every cellular context; concentration, tissue exposure, metabolic state, and cancer versus non-cancer biology matter, as illustrated by work showing that SCFAs can modulate iron metabolism and ferroptosis in cancer cells [55]. Bile acids occupy a similar dual role. They are detergents capable of membrane and mitochondrial injury when excessive or hydrophobic, yet they are also endocrine signals through FXR and TGR5 that regulate lipid metabolism, glucose metabolism, inflammation, and mitochondrial energy balance. Dysregulated bile acid metabolism has been linked to lipid peroxidation and ferroptosis in MASLD, while ALDH2-deficiency and cholestasis-related models implicate gut microbiota dysbiosis and altered bile acid metabolism in liver or placental redox injury [56,57,58]. Tryptophan-derived indoles can activate aryl hydrocarbon receptor signaling, preserve barrier function, and modulate inflammation, whereas dysregulated tryptophan metabolism may contribute to immune and vascular dysfunction. The framework is therefore best viewed as a metabolite balance problem: cardiometabolic risk increases when microbial ecology shifts from barrier-supporting, mitochondrial-compatible signaling toward endotoxemic, pro-inflammatory, and lipid-peroxidizing states.

### 3.3. Microbial Metabolites as Mitochondrial and Ferroptosis Modulators

Microbial metabolites can influence ferroptosis through several non-mutually exclusive routes. First, they can regulate mitochondrial fuel selection. SCFAs and bile acids may alter AMPK, PPAR, FXR, TGR5, and sirtuin-linked pathways, thereby changing fatty acid oxidation, respiratory efficiency, and mitochondrial antioxidant capacity. Second, they can influence cysteine, methionine, and glutathione metabolism, which directly affects the SLC7A11-GSH-GPX4 axis. Third, they can remodel lipid availability and phospholipid composition, changing the pool of polyunsaturated fatty acid-containing phospholipids susceptible to peroxidation. Fourth, they can regulate iron absorption, ferritin turnover, heme metabolism, and inflammatory iron sequestration, as suggested by iron overload and hemochromatosis-related models linking gut microbiota, ROS, and ferroptosis [14,59,60]. Finally, they can shape immune cell metabolism, which influences whether macrophages amplify oxidative injury or support resolution.

The same metabolite class may therefore have protective and injurious effects depending on context. Butyrate can support epithelial mitochondrial respiration and barrier integrity, but excessive or ectopic exposure may alter cellular proliferation and redox tone. Bile acids can improve metabolic signaling through receptor-mediated effects, yet hydrophobic bile acid accumulation may damage mitochondria, increase ROS, and sensitize hepatocytes to lipid peroxidation. TMAO may mark microbial–host co-metabolism and renal clearance as much as direct toxicity, but in vascular and renal contexts it remains a useful signal of diet–microbiome–host interaction. These examples argue against a simple “good metabolite versus bad metabolite” classification. A more useful approach is to ask whether a metabolite pattern preserves mitochondrial redox flexibility and lipid peroxide repair in a given tissue, and whether the evidence comes from association, causal manipulation, or human intervention.

**Table 2:** Gut-derived microbial metabolites involved in mitochondrial redox imbalance and ferroptosis.

Microbial Signal	Main Source/Origin	Redox-Related Effect	Ferroptosis-Related Effect	Cardiometabolic Relevance
LPS	Gram-negative bacteria; barrier leakage	Activates TLR4/NF-kappaB; increases inflammatory ROS; impairs mitochondrial function	Promotes lipid peroxidation and inflammatory ferroptosis through Nrf2/GPX4/SLC7A11-sensitive pathways	Atherosclerosis, obesity, insulin resistance, MASLD, CKD [31,32,33,34,41]
TMAO	Microbial choline/carnitine metabolism followed by hepatic oxidation	Associated with oxidative stress, macrophage lipid accumulation, endothelial dysfunction; causal strength varies in humans	May enhance lipid peroxidation and iron-related injury in vascular or renal contexts	Atherosclerosis, thrombosis, heart failure, CKD; influenced by diet and renal clearance [47,48,49,50,51,52,53]
SCFAs	Fermentation of dietary fiber and resistant starch	Usually reduces oxidative stress, supports barrier integrity, and improves mitochondrial metabolism	Generally protects redox balance; context-dependent effects on iron metabolism and ferroptosis require caution	Obesity, diabetes, vascular dysfunction, gut–liver metabolic disease [35,54,55,61]
Bile acids	Gut–liver bile acid transformation	Regulate mitochondrial metabolism, FXR/TGR5 signaling, lipid handling, and oxidative stress	Alter lipid substrate availability and ferroptotic susceptibility in steatotic liver disease	MASLD, obesity, insulin resistance [56,57,58]
Tryptophan metabolites	Microbial tryptophan metabolism	Regulate AhR signaling, barrier function, inflammation, and mitochondrial stress	May influence antioxidant defense, immune tone, and lipid peroxidation susceptibility	Diabetes, MASLD, vascular inflammation [62,63]

## 4. Mitochondrial Redox Dysfunction in Cardiometabolic Disease

Mitochondria are not only sources of ATP but also hubs of redox signaling, lipid metabolism, calcium handling, innate immune activation, apoptosis, and ferroptosis sensitivity. In cardiometabolic tissues, nutrient overload increases electron pressure on the respiratory chain, raises mtROS production, promotes incomplete fatty acid oxidation, disrupts mitochondrial membrane potential, and alters the NADH/NAD+ and GSH/GSSG redox couples. These changes are amplified by insulin resistance, hypoxia, ER stress, iron accumulation, and inflammatory cytokines. In MASLD, recent reviews frame mitochondrial autophagy and mitochondrial quality control as important contributors to pathogenesis [15,26,27]. In vascular disease, endothelial mitophagy and mitochondrial fission have been linked to inflammatory and oxidative damage in atherosclerosis models, while broader cardiotoxicity and cardiometabolic models increasingly connect mitochondrial stress to ferroptosis-sensitive injury [37,64,65]. The result is a transition from adaptive mitochondrial signaling to maladaptive mitochondrial redox dysfunction.

Mitochondrial quality control influences whether this stress is repaired or propagated. Fusion can dilute focal damage, fission can isolate damaged organelles, mitophagy can remove dysfunctional mitochondria, and biogenesis can restore energetic reserve. In MASLD, diabetic cardiomyopathy, vascular dysfunction, and kidney disease, defective mitophagy and imbalanced mitochondrial dynamics often coexist with mtROS accumulation and impaired oxidative phosphorylation [15,26,66,67,68]. Mitochondrial DNA released from damaged mitochondria can activate cGAS-STING and other innate immune pathways, linking organelle injury to sterile inflammation. Meanwhile, mitochondrial iron handling and cardiolipin-rich membranes can influence ferroptosis susceptibility, especially when lipid peroxide detoxification systems are overwhelmed.

The mitochondrial contribution to ferroptosis is context-dependent but biologically important in cardiometabolic tissues. In cells that depend heavily on oxidative phosphorylation, mitochondrial membrane potential, respiratory chain activity, and tricarboxylic acid cycle flux can influence the rate of ROS generation and lipid peroxide amplification. Mitochondria also shape NADPH availability, cysteine metabolism, iron–sulfur cluster biogenesis, heme synthesis, and CoQ redox cycling. When nutrient overload, inflammatory signaling, and hypoxia converge, mitochondria may shift from metabolic buffers to lipid peroxidation amplifiers. This is particularly relevant for cardiomyocytes and renal tubular cells, which have high energetic demand, and for hepatocytes, which coordinate lipid and iron metabolism [41,59,69,70,71].

**Figure 2:**
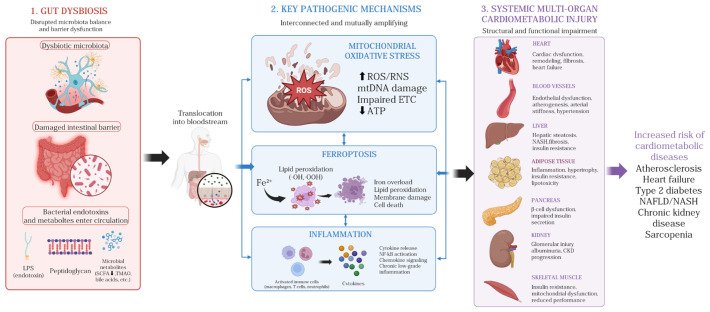
Mechanistic view of the gut microbiota–mitochondria ferroptosis framework. Gut dysbiosis, altered microbial metabolites, and barrier leakage converge on mitochondrial redox dysfunction, impaired mitophagy, mtDNA release, iron-dependent lipid peroxidation, GPX4/SLC7A11 suppression, and inflammatory signaling. The figure highlights the four major checkpoints discussed in the text: gut barrier and metabolite exposure, mitochondrial quality control, lipid peroxide detoxification, and inflammatory amplification. Created in BioRender. Mingyuan, L. (2026) https://BioRender.com/hrzvenc, accessed on 24 May 2026.

Mitophagy provides an adaptive brake on this process. Efficient removal of damaged mitochondria can limit mtROS, reduce cytosolic mtDNA accumulation, and preserve metabolic flexibility. However, excessive or defective mitophagy can both be harmful: insufficient mitophagy leaves damaged organelles in place, whereas excessive mitochondrial turnover may deplete bioenergetic capacity. In cardiometabolic disease, mitophagy should therefore be interpreted together with mitochondrial biogenesis, respiratory function, and cell survival rather than as a universally beneficial marker. Redox-targeted interventions that activate AMPK, SIRT3, PINK1/Parkin-related pathways, or Nrf2 may act partly by restoring this quality control balance; examples include L-citrulline effects on AMPK-linked mitochondrial quality control and iron metabolism, and studies connecting mitochondrial function, ferritinophagy, and gut microbiota remodeling [59,60], detailed in Figure 2.

## 5. Ferroptosis as a Redox-Sensitive Injury Mechanism

Ferroptosis is defined by iron-dependent phospholipid peroxidation and failure of lipid peroxide repair. Its core protective machinery includes system x_c_^−^, composed of SLC7A11 and SLC3A2, which imports cystine for glutathione synthesis; glutathione peroxidase 4 (GPX4), which reduces phospholipid hydroperoxides; and parallel systems such as FSP1/CoQ10 and DHODH that suppress lipid radical propagation. Pro-ferroptotic mechanisms include labile iron accumulation, ferritinophagy, ACSL4-mediated incorporation of polyunsaturated fatty acids into phospholipids, lipoxygenase activity, mitochondrial dysfunction, and depletion of GSH. Cardiometabolic diseases create a favorable environment for ferroptosis because they combine lipid abundance, oxidative pressure, inflammation, and disturbed iron metabolism. In MASLD, liproxstatin-1 reduced lipid peroxidation and ferroptosis markers in experimental disease; in myocardial ischemia–reperfusion and doxorubicin cardiotoxicity, lipid metabolism and cysteine redox state have been linked to ferroptosis-sensitive cardiac injury; and in diabetic nephropathy, ferroptosis and NLRP3-related inflammatory injury have been linked to mitochondrial damage [10,11,13,72].

The ferroptosis concept is especially useful because it connects classical oxidative stress markers with regulated cell death. Malondialdehyde and 4-hydroxynonenal are not just passive damage markers; they reflect lipid peroxide chemistry that can modify proteins, impair mitochondrial enzymes, alter membrane properties, and activate inflammatory responses. In endothelial cells, ferroptosis-sensitive injury may reduce nitric oxide signaling, increase permeability, and contribute to plaque progression. In cardiomyocytes, ferroptosis has been implicated in ischemia–reperfusion injury, diabetic cardiomyopathy, septic cardiomyopathy, and drug-induced cardiotoxicity [13,42,71,72,73,74]. In hepatocytes, ferroptosis has been linked to steatohepatitis, toxic injury, and fibrosis [10,75,76,77,78]. In renal tubular cells and podocytes, it has been implicated in diabetic kidney disease, acute kidney injury, and cardiorenal metabolic stress [11,33,40,79,80,81]. These tissue-specific manifestations share a redox grammar: iron plus susceptible lipids plus insufficient peroxide repair.

Several features make ferroptosis especially compatible with cardiometabolic pathology. First, cardiometabolic tissues are exposed to lipid overload, creating abundant substrates for peroxidation. Second, metabolic inflammation changes iron distribution by increasing hepcidin, ferritinophagy, macrophage iron retention, or labile iron release depending on tissue context. Third, mitochondrial overload and NADPH depletion weaken endogenous repair systems. Fourth, inflammatory cytokines can suppress antioxidant genes, alter amino acid transport, and increase lipid-remodeling enzymes. Finally, ferroptotic cells release oxidized lipids and damage-associated signals that recruit immune cells. Ferroptosis therefore should not be viewed only as a terminal cell death label; it is a biochemical state that links membrane lipid composition, iron metabolism, mitochondrial redox, and inflammation. In human-facing work, however, the term should be used cautiously unless several convergent markers are measured; many studies currently support “ferroptosis-sensitive” or “ferroptosis-like” injury rather than definitive ferroptotic cell death in patient tissues.

The main endogenous anti-ferroptotic systems provide a useful map for intervention. The SLC7A11-GSH-GPX4 pathway supports phospholipid protection by maintaining glutathione-dependent peroxide reduction; endothelial and LPS-related studies have shown that SLC7A11/GPX4 regulation can shape ferroptosis and inflammation [12,39,82,83]. The FSP1-CoQ10 pathway suppresses lipid radical propagation at the membrane independently of GPX4. The mitochondrial DHODH-CoQ system is especially relevant when mitochondrial lipid peroxidation contributes to cell death, and DHODH-related regulation has been examined in myocardial injury models [69]. Nrf2 coordinates many protective genes, including those involved in glutathione synthesis, iron sequestration, heme metabolism, and detoxification; several recent models report Nrf2/HO-1 or Nrf2/GPX4-centered protection across inflammatory, renal, and cardiotoxic settings [40,68,74,81,84,85]. These systems are interconnected rather than redundant: if cysteine supply is limited, GPX4 activity falls; if mitochondrial CoQ redox cycling is impaired, local lipid peroxide burden may rise; if iron is not properly stored, Fenton chemistry accelerates lipid radical formation. Antioxidants that support these endogenous systems may be more rational than direct radical scavengers with uncertain tissue distribution.

## 6. Crosstalk Among Gut Dysbiosis, Mitochondrial Injury, Ferroptosis, and Inflammation

The gut microbiota–mitochondria ferroptosis framework is not linear in a simple sense. It is a reinforcing loop. Dysbiosis increases LPS and metabolite imbalance, which activate inflammatory receptors and alter mitochondrial metabolism. Mitochondrial dysfunction increases mtROS and mtDNA release, which activate NLRP3 inflammasome and cGAS-STING signaling. Ferroptosis-sensitive lipid peroxidation produces electrophilic aldehydes and oxidized phospholipids that further stimulate inflammation. Inflammation then worsens barrier dysfunction, insulin resistance, endothelial activation, and mitochondrial stress. Experimental evidence from diabetic nephropathy, LPS myocardial injury, acute kidney injury, septic cardiomyopathy, and inflammatory organ injury illustrates how ferroptosis-sensitive injury, mitochondrial damage, Nrf2 signaling, and inflammasome activity can reinforce one another in metabolically relevant tissues [11,34,42,71,86,87]. Thus, the framework can maintain itself even after the initiating insult has diminished.

A useful way to conceptualize this loop is to distinguish four redox checkpoints. The first is the gut barrier checkpoint, where mucus, tight junctions, antimicrobial peptides, and microbial ecology shape systemic exposure to pro-inflammatory products. The second is the mitochondrial checkpoint, where nutrient flux, OXPHOS efficiency, mitophagy, and antioxidant buffering influence whether microbial and metabolic signals become adaptive or damaging. The third is the ferroptosis checkpoint, where iron handling, GPX4/SLC7A11 activity, CoQ10-dependent radical trapping, and lipid composition influence whether lipid peroxides remain repairable. The fourth is the inflammatory checkpoint, where NF-kappaB, NLRP3, cGAS-STING, macrophage polarization, and cytokine networks influence whether injury resolves or progresses [11,12,39,40,69].

**Figure 3:**
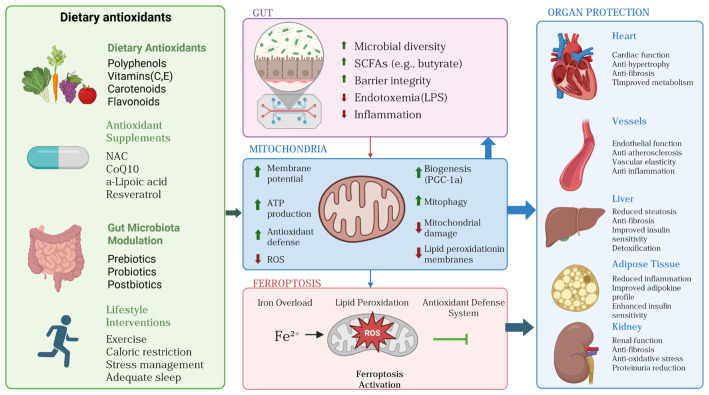
Redox-targeted intervention concepts within the proposed gut microbiota–mitochondria ferroptosis framework. Natural antioxidants, microbiota-targeted therapies, mitochondria-targeted antioxidants, ferroptosis modulators, and nutritional strategies may engage microbial, mitochondrial, lipid peroxide, or inflammatory checkpoints. The figure summarizes candidate mechanisms and should not be interpreted as showing equivalent evidence or uniformly beneficial effects for all intervention classes; much of the mechanism-specific evidence remains preclinical. Successful translation will require evidence of exposure, target engagement, mechanism-specific biomarkers, and clinically meaningful benefit. Created in BioRender. Mingyuan, L. (2026) https://BioRender.com/3zt7xxq, accessed on 24 May 2026.

These checkpoints are clinically relevant because they suggest different intervention windows. In early metabolic dysfunction, dietary fiber, exercise, weight reduction, and microbiota-supportive diets may primarily improve the gut barrier and metabolite profile like Figure 3. In established insulin resistance or MASLD, mitochondrial and lipid-metabolic interventions may be required to reduce tissue-level redox pressure. In advanced vascular, hepatic, renal, or cardiac injury, ferroptosis-sensitive and anti-inflammatory approaches may be needed to prevent irreversible cell loss and fibrosis. A precision strategy would therefore define where a patient sits along the framework rather than applying the same antioxidant regimen to all cardiometabolic phenotypes.

**Table 3:** Key molecular pathways linking gut dysbiosis, mitochondrial dysfunction, ferroptosis, and inflammation.

Pathway/Molecule	Biological Role	Relationship with Mitochondrial Redox	Relationship with Ferroptosis	Potential Intervention Point
Nrf2/HO-1	Endogenous antioxidant and detoxification defense	Enhances redox buffering and limits ROS amplification	Regulates GPX4, GSH metabolism, and iron handling	Polyphenols, melatonin, sulforaphane-like Nrf2 activators [39,40]
GPX4/GSH	Lipid peroxide detoxification	Preserves membrane and mitochondrial redox homeostasis	Core anti-ferroptotic pathway; loss increases lipid peroxide death	GPX4 preservation, GSH restoration, NAC-like cysteine support [10,11]
SLC7A11/xCT	Cystine uptake for GSH synthesis	Supports intracellular thiol buffering	Suppression promotes ferroptosis through GSH depletion	SLC7A11 restoration, Nrf2 activation [12,39]
ACSL4	Incorporates PUFAs into membrane phospholipids	Increases oxidizable lipid substrates under redox stress	Promotes ferroptotic lipid peroxidation	ACSL4/lipid-remodeling inhibition [10,13]
FSP1/CoQ10	Non-GPX4 lipid radical-trapping system	Supports membrane redox protection	Suppresses ferroptosis independently of GPX4	CoQ10-related strategies, FSP1 pathway support [29,30]
DHODH/CoQ	Mitochondrial pyrimidine and CoQ redox coupling	Modulates mitochondrial electron transfer and local antioxidant capacity	Restrains mitochondrial lipid peroxidation	DHODH/CoQ-targeted mitochondrial protection [29,69]
PINK1/Parkin	Mitophagy and mitochondrial quality control	Removes damaged mitochondria and lowers mtROS	May reduce mtROS-driven ferroptosis susceptibility	Mitophagy activators, AMPK-linked strategies [15,59]
NLRP3 inflammasome	Inflammatory activation	Activated by mtROS, mitochondrial damage, and ionic stress	Amplifies ferroptosis-associated inflammatory injury	MCC950-like inhibitors, polyphenols, probiotics [11,84]
cGAS-STING	mtDNA-triggered innate immunity	Activated by mitochondrial damage and mtDNA release	Links organelle injury to inflammatory cell death programs	STING inhibition, mitochondrial stabilization [68,70]
NF-kappaB	Pro-inflammatory transcription	Activated by ROS, LPS, and cytokine signaling	Promotes cytokine-linked ferroptotic susceptibility	Curcumin, resveratrol, microbiota modulation [22,31,88]

## 7. Disease-Specific Evidence

### 7.1. Atherosclerosis and Vascular Dysfunction

Atherosclerosis is a prototypical disease in which gut-derived metabolites, mitochondrial dysfunction, lipid oxidation, and inflammation converge. TMAO and other microbial products have been linked to cholesterol handling, macrophage foam cell formation, endothelial activation, and platelet responses. In macrophage foam cells, TMAO was reported to promote oxidative stress and lipid accumulation through Nrf2/ABCA1-related pathways, providing a direct metabolite-to-vascular-redox example [47]. At the same time, human evidence is not uniform: reviews and meta-analyses support clinical associations, whereas Mendelian randomization studies are less consistent for causality [49,50,52,53]. Endothelial mitochondria are vulnerable to hyperlipidemia, hyperglycemia, disturbed flow, and inflammatory cytokines, and mitochondrial dysfunction can reduce nitric oxide bioavailability while increasing mtROS. Ferroptosis adds an additional layer: iron accumulation and lipid peroxidation in endothelial cells, macrophages, and vascular smooth muscle cells may destabilize plaques, impair endothelial repair, and amplify inflammatory signaling. Anti-atherogenic activities have been reported for exopolysaccharides and their producing probiotic strain, while multi-omics work on ginsenoside Rb1, baicalin, and mechanistic reviews of emerging atherosclerosis risk factors support a broader microbiota redox intervention space [5,20,43,89,90].

Within plaques, the framework may operate at several cellular levels. Endothelial dysfunction increases monocyte recruitment and vascular permeability; macrophage mitochondrial stress favors inflammatory polarization and foam cell persistence; smooth muscle cell phenotypic switching affects fibrous cap stability; and ferroptosis-like lipid peroxidation may expand necrotic cores. Gut-derived metabolites can modulate each step through cholesterol efflux, bile acid pools, platelet activation, and vascular inflammation. This multi-cellular setting helps explain why purely lipid-lowering therapy, while essential, may not fully resolve residual inflammatory and redox risk. Microbiota-informed and ferroptosis-informed biomarkers could help identify patients in whom residual vascular risk is associated with metabolite imbalance, mitochondrial dysfunction, or lipid peroxide burden. Overall, the evidence in atherosclerosis is mechanistically coherent and supported by animal, cellular, multi-omics, and clinical association studies, but it is best classified as mainly Level 1–2 evidence with selected Level 3 preclinical support; complete Level 4 human chain evidence is lacking. TMAO should be interpreted as a diet–microbiome–host co-metabolic marker and possible disease modifier rather than as a universally causal toxin, especially because circulating levels are influenced by renal function, diet, host metabolism, and microbiome composition.

### 7.2. Diabetes, Insulin Resistance, and Diabetic Cardiomyopathy

Type 2 diabetes and insulin resistance generate redox stress through nutrient surplus, glucolipotoxicity, advanced glycation, mitochondrial overload, and chronic inflammation. Reviews of oxidative stress biomarkers in hypertension and diabetes, cellular pathophysiology in gestational diabetes, and therapeutic strategies for type 2 diabetes all emphasize that redox imbalance is tightly coupled to metabolic and inflammatory remodeling [1,19,91,92]. Dysbiosis may worsen this state by reducing barrier function, changing bile acid and SCFA signaling, increasing endotoxemia, and modifying incretin and immune pathways. In diabetic cardiomyopathy, cardiomyocytes face high fatty acid flux, impaired mitochondrial flexibility, mtROS accumulation, calcium mishandling, and defective mitophagy. Paeoniflorin has been reported to confer ferroptosis resistance through gut microbiota and metabolites in diabetic cardiomyopathy, making it one of the more direct pieces of evidence for this framework in the diabetic heart [66]. A particularly attractive therapeutic hypothesis is that microbiota-directed interventions can reduce upstream inflammatory and metabolic pressure while antioxidants or ferroptosis modulators protect cardiomyocytes from downstream lipid peroxide death.

Diabetic cardiomyopathy illustrates the difference between metabolic stress and ischemic injury. Even without obstructive coronary disease, the diabetic heart can develop energetic inflexibility, mitochondrial calcium imbalance, impaired mitophagy, interstitial fibrosis, and diastolic dysfunction. Hyperglycemia and high fatty acid flux increase mitochondrial electron pressure, while systemic inflammation and gut-derived endotoxemia lower the threshold for inflammatory activation. Ferroptosis-associated injury may be particularly important when lipid overload and iron dysregulation coincide with weakened GPX4 activity. In this context, antioxidant and redox-targeted strategies should be judged by whether they improve cardiac substrate flexibility, preserve mitochondrial dynamics, prevent lipid peroxide accumulation, and reduce inflammatory remodeling. Evidence is strongest when cardiac function, microbial metabolites, mitochondrial phenotypes, and lipid peroxidation markers move together; evidence is weaker when only serum oxidative stress markers or taxonomic shifts are reported. Overall, evidence for a gut microbiota–mitochondria ferroptosis framework in diabetes and diabetic cardiomyopathy is promising but remains dominated by animal and cellular studies. The strongest preclinical work approaches Level 3 evidence, whereas longitudinal cohorts and intervention trials that test microbial metabolites, cardiac function, and ferroptosis-sensitive biomarkers together are still needed.

### 7.3. MASLD and the Gut–Liver Axis

MASLD is perhaps the clearest disease context for this framework because the liver receives gut-derived metabolites through the portal circulation and is central to lipid, bile acid, glucose, and iron metabolism. Dysbiosis and intestinal permeability contribute to hepatic LPS exposure, TLR4 activation, Kupffer cell stimulation, and oxidative stress. Reviews focused on gut microbiota, oxidative stress, and MASLD explicitly highlight gut–liver crosstalk, lipid peroxidation, bile acid remodeling, and ferroptosis [4,6,7,8]. Hepatocyte mitochondria under lipid overload generate mtROS, lose respiratory efficiency, and activate inflammatory and fibrogenic pathways; mitochondrial–autophagy and mitochondrial quality control reviews position these processes as central MASLD mechanisms [15,26]. Ferroptosis contributes to steatohepatitis through iron-dependent lipid peroxidation, ACSL4 activity, GSH depletion, and GPX4 insufficiency, and liproxstatin-1 has been reported to alleviate experimental steatotic liver disease [10]. Multi-omics and intervention studies of Tricholoma mongolicum polysaccharides, Pueraria lobata polysaccharide, Lycium ruthenicum fructan, ginsenoside Rd, Camellia Japonica Radix, propyl gallate, and bile-acid-targeted strategies increasingly point to a gut microbiota–metabolite–ferroptosis circuit in MASLD and hepatic fibrosis models [21,56,75,76,93,94,95].

The disease spectrum from steatosis to steatohepatitis and fibrosis can be interpreted as progressive failure of redox adaptation. In early steatosis, lipid droplets may partly buffer excess fatty acids. With persistent nutrient overload and inflammatory input, mitochondrial beta-oxidation becomes insufficient or inefficient, ER stress rises, and lipid peroxide products accumulate. Kupffer cells and recruited macrophages then intensify cytokine production, while stellate cells respond to oxidized lipids and inflammatory mediators by promoting fibrosis. Ferroptosis-associated hepatocyte injury may contribute both to cell loss and to inflammatory signaling that recruits fibrogenic responses. Therapeutic approaches that improve gut barrier function, normalize bile acid signaling, support mitochondrial oxidation, and preserve GPX4/SLC7A11 activity may therefore be most relevant before irreversible fibrosis dominates. In evidence hierarchy terms, MASLD has richer preclinical, multi-omics, and intervention evidence than several other cardiometabolic contexts, including several Level 3 studies and limited preclinical Level 4-like designs; however, direct demonstration that manipulating the microbiota modifies human hepatic ferroptosis remains limited.

### 7.4. Obesity, Adipose Inflammation, and Metabolic Syndrome

Obesity changes gut microbial composition, increases intestinal permeability, alters bile acid and SCFA profiles, and promotes adipose tissue inflammation. Hypertrophic adipocytes are mitochondrially stressed and release fatty acids, cytokines, and danger signals that recruit macrophages and worsen insulin resistance. Although ferroptosis in adipose tissue is less developed than in liver, heart, and kidney, lipid overload, iron handling, and oxidized phospholipid biology make it mechanistically plausible. Nutrient-based strategies for obesity-related fatty liver disease, flavonoid–microbiome interactions relevant to cardiovascular health, high-fat-diet models testing caffeine–naringenin, and rare-sugar interventions in diabetic metabolic dysfunction support the idea that obesity links diet, microbiota, and redox injury [16,96,97,98]. The more immediate clinical relevance is that obesity supplies the systemic substrate load and inflammatory tone that sensitize cardiometabolic organs to mitochondrial redox imbalance and ferroptosis-sensitive injury.

Adipose tissue also acts as an endocrine redox organ. As adipocytes enlarge, local hypoxia, ER stress, mitochondrial dysfunction, and macrophage infiltration increase. Adipokine imbalance and free fatty acid spillover then expose liver, skeletal muscle, heart, and endothelium to metabolic stress. Gut microbiota can modulate this process through energy harvest, bile acid signaling, appetite-related peptides, and inflammatory tone. Exercise and dietary patterns may protect partly because they improve mitochondrial oxidative capacity and microbial metabolite profiles at the same time, reducing the upstream pressure that sensitizes distant organs to ferroptosis-sensitive injury. The adipose component of the framework should nevertheless be interpreted as an amplifier of systemic risk rather than as the best-established ferroptotic target tissue. Overall, the obesity evidence is more indirect than the hepatic, renal, or cardiac evidence and is mostly Level 1–2: human data largely support microbiome–metabolic associations, whereas adipose tissue ferroptosis and its modification by microbiota-directed interventions require stronger causal and interventional validation.

### 7.5. Chronic Kidney Disease and Cardiorenal Metabolic Syndrome

The kidney is exposed to circulating microbial metabolites, uremic toxins, hyperglycemia, hypertension, and inflammatory mediators. Dysbiosis can both result from and contribute to chronic kidney disease through altered nitrogen metabolism, gut barrier dysfunction, TMAO accumulation, and systemic inflammation. Reviews of CKD metabolic abnormalities and diabetic kidney disease provide the broader clinical frame, while probiotic-focused work highlights gut microbiota modulation as a potential strategy in diabetic kidney disease [2,24,25]. Renal tubular cells have high mitochondrial demand and are vulnerable to mtROS, iron accumulation, and lipid peroxidation. Ferroptosis-associated renal injury has been implicated in diabetic kidney disease, acute kidney injury, and inflammatory renal injury through Nrf2, GPX4, SLC7A11, ferritinophagy, and mitochondrial pathways [11,33,40,81]. Gut–kidney intervention studies using nobiletin and morroniside support a microbiota–redox–kidney framework [79,80]. Additional work on 2′-fucosyllactose, astragaloside IV, and food medicine homologous herbs extends this concept to hyperuricemia, nephrotoxicity, and diabetic kidney disease contexts [99,100,101]. Overall, CKD and diabetic kidney disease have strong Level 2–3 preclinical support for mitochondrial injury and ferroptosis-associated injury, plus clinical association evidence for dysbiosis and microbial metabolites; however, kidney function is itself a major confounder and effect modifier, especially for TMAO and other renally cleared microbial products.

**Figure 4:**
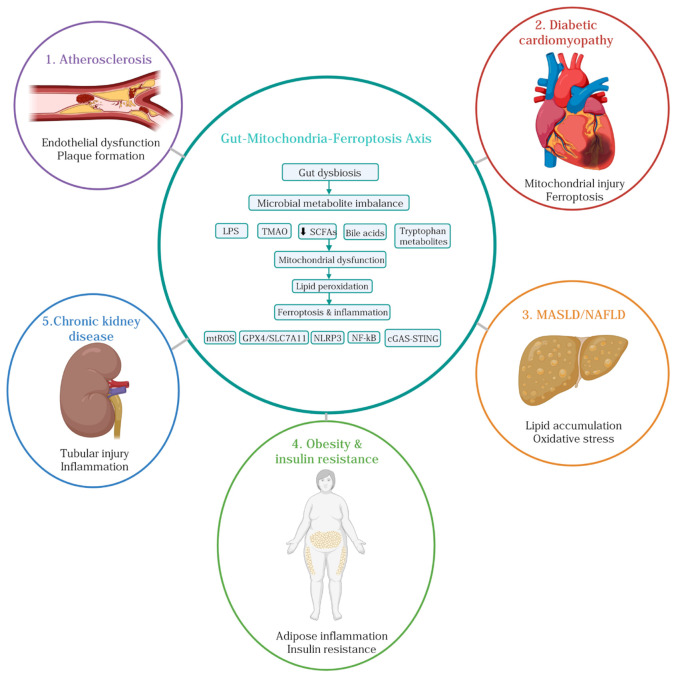
Disease contexts in which components of the proposed gut microbiota–mitochondria ferroptosis framework have been reported. Atherosclerosis, diabetic cardiomyopathy, MASLD, obesity-associated insulin resistance, chronic kidney disease, and cardiorenal metabolic injury differ in tissue manifestation but share microbial metabolite imbalance, mitochondrial redox stress, ferroptosis-sensitive lipid peroxidation, and inflammation. The figure is a disease-context map rather than an evidence-strength heat map; the graded evidence summary is provided in Table 4. Created in BioRender. Mingyuan, L. (2026) https://BioRender.com/568bpy9, accessed on 24 May 2026.

Cardiorenal metabolic syndrome highlights feedback across organs. Declining kidney function increases circulating uremic solutes and microbial metabolites, which can worsen vascular calcification, endothelial dysfunction, oxidative stress, and cardiac remodeling. At the same time, heart failure and vascular disease impair renal perfusion and intensify renal mitochondrial injury. Ferroptosis-sensitive tubular damage may therefore amplify systemic cardiometabolic risk rather than remaining a local renal event. Microbiota-targeted strategies in kidney disease should be assessed not only by renal endpoints but also by vascular inflammation, cardiac function, and circulating lipid peroxide or iron-related markers. Because renal clearance strongly influences TMAO and other microbial metabolites, kidney function should also be treated as a confounder and effect modifier in cardiometabolic microbiome studies as shown in Figure 4. Thus, cardiorenal evidence should be read as a combination of animal/cell mechanism, clinical association, and biologically plausible cross-organ feedback rather than as proof that any single microbial metabolite initiates the syndrome.

**Table 4:** Disease-specific involvement and evidence strength of the gut microbiota–mitochondria ferroptosis framework in cardiometabolic disorders.

Disease	Gut-Related Changes	Mitochondrial Redox Abnormality	Ferroptosis-Related Features	Inflammatory Component	Level	Main Evidence Type
Atherosclerosis and vascular dysfunction	Dysbiosis, increased TMAO and LPS exposure	Endothelial mtROS, mitochondrial dysfunction, impaired mitophagy	Lipid peroxidation and iron-related injury; endothelial/macrophage ferroptosis-associated phenotypes	NF-kappaB, NLRP3, macrophage activation	1–3	Animal/cell and clinical association; incomplete human causal chain [43,47,64]
Diabetic cardiomyopathy	Dysbiosis and altered microbial metabolites	Cardiomyocyte mtROS, impaired metabolic flexibility, defective mitophagy	Ferroptosis-associated cardiomyocyte injury; GPX4/SLC7A11 disturbance	NLRP3, IL-1beta, TNF-alpha	2–3	Mainly animal and cell models; limited human biomarker integration [1,23,66]
MASLD	Barrier injury, LPS exposure, bile acid imbalance	Hepatic mitochondrial dysfunction, mtROS, impaired quality control	Hepatocyte lipid peroxidation, ACSL4 increase, GPX4 loss, iron overload	Kupffer cell activation, TLR4/NF-kappaB	2–4	Animal, omics, and intervention evidence; human complete-chain evidence limited [6,7,10,21,75]
Obesity and insulin resistance	Reduced diversity, altered SCFAs, barrier dysfunction	Adipocyte and hepatic mitochondrial stress	Lipid peroxide accumulation; ferroptosis-like susceptibility in target organs	Macrophage infiltration, chronic low-grade inflammation	1–2	Animal and clinical association; adipose ferroptosis remains indirect [54,96,97]
CKD and cardiorenal syndrome	Uremic dysbiosis, barrier dysfunction, microbial toxin accumulation	Renal tubular mtROS, mitochondrial energy stress	Tubular ferroptosis-associated injury, iron overload, GPX4 loss	NLRP3, cytokine release, complement activation	2–3	Animal, cell, and clinical association; renal clearance confounds metabolites [2,11,25,79]

## 8. Redox-Targeted Interventions

### 8.1. From ROS Scavenging to Redox Network Modulation

The mixed clinical history of antioxidants reflects a conceptual problem: cardiometabolic disease is not caused by a uniform pool of ROS that can be neutralized by generic scavengers. ROS participate in insulin signaling, mitochondrial adaptation, vascular tone, immune defense, and exercise responses, while reactive nitrogen and sulfur species also participate in vascular and metabolic signaling. Excessive suppression may blunt adaptive signaling, whereas insufficiently targeted antioxidant therapy may fail to reach the relevant compartment or lipid domain. Large randomized trials of vitamin E and vitamin C generally did not show consistent cardiovascular benefit, and meta-analytic evidence for broad antioxidant vitamin supplementation remains disappointing [102,103,104,105,106]. The inconsistent performance of antioxidant interventions in cardiometabolic trials may reflect several factors, including poor tissue delivery, inadequate baseline redox phenotyping, heterogeneous background medication, microbiome-dependent metabolism, non-specific ROS suppression, and endpoints that do not confirm target engagement. These negative or neutral findings do not invalidate redox biology; they show that nonselective antioxidant supplementation, without target engagement, exposure verification, or patient stratification, is unlikely to correct complex cardiometabolic networks. Modern redox-targeted strategies should therefore be evaluated by whether they restore redox networks: mitochondrial function, GSH/GPX4 activity, Nrf2-regulated cytoprotection, lipid peroxide repair, iron homeostasis, barrier integrity, and inflammatory resolution. This network view is consistent with reviews of phytochemicals and flavonoids in cardiovascular health, oxidative-stress biomarker reviews in diabetes and hypertension, and probiotic-focused antioxidant strategies in diabetes [1,16,17,23].

A practical classification is to divide interventions by their dominant checkpoint. Gut-directed interventions aim to reduce endotoxemia and improve metabolite balance. Mitochondria-directed interventions aim to restore respiratory efficiency, mitophagy, and organelle antioxidant capacity. Ferroptosis-directed interventions aim to preserve lipid peroxide repair and iron homeostasis. Inflammation-directed interventions aim to prevent redox damage from becoming self-sustaining. Many natural products and nutraceuticals cross these categories, which is a strength for network diseases but a challenge for trial design. Mechanistic readouts should therefore be built into intervention studies, including microbial metabolite panels, mitochondrial function assays, lipidomics, iron markers, inflammatory biomarkers, and exposure markers that distinguish parent compounds from microbiota-derived antioxidant metabolites. A successful framework-guided intervention should meet at least four criteria: adequate exposure of the active compound or microbial metabolite, measurable engagement of the intended checkpoint, improvement in disease-relevant functional or clinical outcomes, and preservation of physiological redox signaling rather than indiscriminate ROS suppression.

### 8.2. Polyphenols, Flavonoids, and Nutraceuticals

Polyphenols and flavonoids are attractive because they can act on multiple nodes: microbial ecology, gut barrier function, bile acid metabolism, AMPK, Nrf2, NF-kappaB, mitochondrial biogenesis, lipid metabolism, and ferroptosis-related pathways. The clearest cardiometabolic examples in the retrieved literature include ginsenoside Rb1 in atherosclerosis-related multi-omics work, ginsenoside Rd in metabolism-associated fatty liver disease, Camellia Japonica Radix in MASLD, and propyl gallate in diabetic liver injury [20,93,94,95]. Tricholoma mongolicum polysaccharides, Pueraria lobata polysaccharide, and Lycium ruthenicum fructan provide food-derived or natural-product examples built around the gut microbiota–metabolite–ferroptosis framework [21,75,76]. Curcumin-related clinical or translational evidence is represented by dialysis and cardiovascular risk studies, while flavonoid-rich fruit extracts, green tea powders, baicalin, fisetin, and phloretin illustrate gut–liver, vascular, and ferroptosis-targeted anti-inflammatory mechanisms [22,83,89,107,108,109,110]. Their effects are often not reducible to direct radical scavenging; many are converted by microbiota into bioactive metabolites, reshape microbial composition, activate endogenous antioxidant defenses, or modulate GPX4/SLC7A11 and lipid peroxidation. For review and trial design, this argues for describing antioxidant candidates by chemical class, source, bioavailability, microbial metabolism, redox target, and disease context rather than only by their nominal total antioxidant capacity.

The microbiome is central to the pharmacology of many polyphenols. Poor absorption in the upper intestine means that substantial amounts reach the colon, where microbial enzymes generate smaller phenolic acids and other metabolites that may be more bioavailable than the parent compounds. These metabolites can feed back on microbial composition, barrier function, bile acid metabolism, and host redox signaling. This creates a circular relationship: baseline microbiota affects polyphenol metabolism, and polyphenol exposure reshapes the microbiota. For review articles and trials, this means that naming a compound is not enough; formulation, dose, food matrix, duration, host diet, and microbial metabolic capacity should be considered part of the intervention. Nano-delivery and metabolite-focused pharmacology may improve exposure, but they also create new questions about tissue distribution, safety, and whether the delivered species matches the biologically active form [27,77].

### 8.3. Microbiota-Targeted Strategies

Probiotics, prebiotics, postbiotics, dietary fibers, fermented foods, and fecal microbiota-related approaches aim to modify upstream inputs of the framework. Potential mechanisms include strengthening the intestinal barrier, reducing LPS translocation, increasing SCFA production, improving bile acid signaling, generating antioxidant metabolites, reducing uremic or pro-atherogenic metabolites, and modulating immune tone. The retrieved evidence includes probiotic strategies for diabetic kidney disease and diabetes complications [23,24]. Anti-atherogenic exopolysaccharides and probiotic strains, Lactobacillus and citicoline in steatohepatitis, and Lactobacillus-mediated protection in myocardial ischemia–reperfusion injury provide organ-specific examples [43,44,111]. Dietary or postbiotic examples include dehulled adlay in fatty liver disease, fermentation-derived products in high-fat-diet-fed mice, postbiotic supplementation in a high-fat-diet model, fecal microbiota transplantation in heat-induced intestinal injury, and screening of lactic acid bacterial strains for anti-inflammatory and antioxidative features [45,46,54,61,112]. In diabetes, MASLD, kidney disease, and vascular dysfunction, microbiota-targeted interventions may be most effective when paired with dietary patterns that sustain ecological change.

Postbiotics and defined microbial metabolites may offer better standardization than live probiotics in some settings. Live organisms must survive manufacturing, storage, gastric acid, bile, and ecological competition; their effects may vary with baseline microbiota. Postbiotics, in contrast, can provide defined cell wall components, secreted molecules, enzymes, or metabolites with more predictable dosing. However, the advantage of live microbes is ecological persistence and substrate-responsive activity. A rational strategy may combine dietary fibers or polyphenols with strains selected for metabolite production, barrier support, or bile acid transformation. For cardiometabolic disease, endpoints should extend beyond taxonomic shifts to include TMAO-related pathways, SCFA profiles, bile acid species, tryptophan metabolites, LPS-binding markers, and tissue redox readouts. Challenges include strain specificity, batch-to-batch viability, antibiotic and medication interactions, diet dependence, and the possibility that the same taxonomic change may produce different metabolite outputs in different hosts.

### 8.4. Mitochondria-Targeted Antioxidants and Ferroptosis Modulators

Mitochondria-targeted antioxidants such as MitoQ, SkQ1-like compounds, CoQ10-related strategies, melatonin, and agents that support NAD+, AMPK, SIRT3, or mitophagy may address the mitochondrial checkpoint more directly than systemic antioxidants. CoQ10 is also mechanistically relevant to ferroptosis because reduced CoQ10 participates in radical-trapping systems, including FSP1-dependent protection. Recent randomized-trial meta-analyses suggest that CoQ10 may improve selected cardiovascular or endothelial function outcomes, but heterogeneity in dose, formulation, background therapy, and endpoints remains substantial [29,30]. Melatonin combines mitochondrial localization, antioxidant effects, anti-inflammatory actions, and potential microbiota interactions. Ferroptosis-focused strategies include GPX4 preservation, SLC7A11 restoration, cysteine/GSH support, iron chelation, ACSL4 modulation, lipoxygenase inhibition, and lipid peroxide trapping. Experimental examples include liproxstatin-1 in experimental steatotic liver disease, higenamine in doxorubicin-induced heart failure through Nrf2/GPX4 signaling, and cysteine imaging identifying a ferroptosis-modulating agent in doxorubicin cardiotoxicity [10,72,73]. Nrf2/GPX4/SLC7A11-centered kidney or cardiac injury models provide additional support for this intervention logic [11,39,40,86].

Mitochondria-targeted compounds also face a delivery paradox. Lipophilic cations can accumulate in polarized mitochondria, but severely depolarized mitochondria may import them less efficiently. In addition, mitochondrial ROS are not uniformly harmful; transient bursts can trigger mitohormesis and adaptive stress resistance. The goal is therefore not to silence mitochondria but to restore redox proportionality: respiratory activity should match substrate load, damaged organelles should be removed, and lipid peroxide repair should remain competent. The same caution applies to ferroptosis inhibitors. Acute inhibition may protect organs during ischemia–reperfusion, toxic injury, or inflammatory flares, whereas chronic systemic suppression could have different effects. Cardiometabolic applications should prioritize tissue-targeted delivery, disease stage stratification, pharmacokinetic measurement, and biomarkers of lipid peroxide burden.

For Table 5, translational evidence was graded before interpretation as follows: Grade A indicates randomized clinical trials with patient-centered clinical outcomes; Grade B indicates human intervention studies with surrogate, pharmacokinetic, or biomarker outcomes; Grade C indicates human observational evidence; and Grade D indicates animal or cell evidence without direct human validation. A range indicates that the category contains heterogeneous agents with different levels of evidence.

**Table 5:** Redox-targeted interventions relevant to the gut microbiota–mitochondria ferroptosis framework, including predefined translational evidence grade and key limitations.

Intervention Category	Representative Agents	Gut-Related Effects	Mitochondrial/Redox Effects	Ferroptosis-Related Effects	Grade	Key Limitations
Polyphenols	Resveratrol, curcumin, anthocyanins, ginsenosides, tea polyphenols	Modulate microbiota and improve barrier function	Activate Nrf2/HO-1, AMPK, mitochondrial quality control	Increase GPX4/SLC7A11; reduce lipid peroxidation and ACSL4-linked injury in models	B–D	Low bioavailability, microbiome-dependent metabolism, heterogeneous formulations and endpoints [20,21,22,75,93]
Flavonoids	Quercetin, fisetin, luteolin, naringenin, baicalin	Improve microbial diversity and metabolite profiles	Activate AMPK/Nrf2; reduce mtROS	Inhibit lipid peroxide propagation and ferroptosis-related inflammation in models	C–D	Dose translation, compound purity, tissue exposure, and limited human ferroptosis readouts [16,83,89,109,110]
Melatonin	Melatonin	May improve barrier function and microbiota balance	Mitochondrial antioxidant; supports mitophagy and respiratory resilience	Reduces iron overload, lipid peroxidation, and GPX4 loss in experimental contexts	B–D	Timing, dose, sleep-related confounding, limited cardiometabolic axis-specific trials
Probiotics	Lactobacillus, Bifidobacterium, exopolysaccharide-producing strains	Restore microbial balance; reduce LPS exposure	Reduce systemic oxidative stress and inflammatory tone	May indirectly reduce ferroptosis-sensitive injury by lowering upstream redox pressure	B–D	Strain specificity, viability, diet dependence, variable colonization, regulatory heterogeneity [23,24,43,44,111,112]
Prebiotics	Inulin, resistant starch, dietary fiber	Increase SCFA production and barrier integrity	Improve mitochondrial substrate handling and metabolic flexibility	Protect redox balance and lipid metabolism; ferroptosis readouts uncommon	B–C	Gastrointestinal tolerance, background diet, responder heterogeneity, limited lipidomics [35,54]
Postbiotics	SCFAs, bacterial metabolites, microbial fractions	Barrier protection and immune modulation	Reduce oxidative stress and improve mucosal redox signaling	Potential anti-ferroptotic effects through improved redox tone	C–D	Standardization, active ingredient definition, dose equivalence, long-term safety [55,61]
Mitochondria-targeted antioxidants	MitoQ, SkQ1, CoQ10, L-citrulline, higenamine	Limited direct gut effect, except indirect microbiota–redox interactions	Improve OXPHOS, mitophagy, CoQ redox cycling, mitochondrial quality control	Reduce mtROS-driven lipid peroxidation in models	B–D	Delivery to damaged mitochondria, formulation variability, disease stage dependence [29,30,59,72,73]
Redox and ferroptosis modulators	NAC-like GSH support, iron chelators, liproxstatin-1, Sestrin2-linked approaches	Variable; may reduce inflammatory spillover secondarily	Restore GSH/Nrf2 balance and iron redox control	Directly inhibit lipid peroxidation and iron-linked ferroptosis in models	D	Mostly experimental; systemic ferroptosis inhibition may affect host defense and tumor biology [10,39,40,86]

## 9. Challenges and Future Perspectives

Several problems limit translation. First, causality remains difficult to establish. Dysbiosis may be an upstream contributor, marker, or consequence of cardiometabolic disease, and many microbial metabolites reflect diet, renal function, medications, and host genetics. Germ-free, antibiotic, fecal transfer, isotope-tracing, organoid, Mendelian randomization, and human intervention studies are needed to move beyond association [49,51,53]. Second, redox interventions are vulnerable to the redox paradox: the same molecule may be antioxidant, pro-oxidant, adaptive, or harmful depending on dose, compartment, disease stage, and local metal or lipid chemistry. Third, ferroptosis biomarkers remain imperfect. Tissue GPX4, ACSL4, 4-HNE, MDA, labile iron, oxidized phosphatidylethanolamines, and transcriptional signatures provide clues, but clinically practical ferroptosis biomarkers are still emerging. Fourth, antioxidant bioavailability and microbiome-dependent metabolism create large inter-individual variation, so pharmacokinetic and metabolite exposure data are essential. Finally, cardiometabolic diseases are multi-organ syndromes; a therapy that improves hepatic steatosis may have different vascular, renal, or cardiac effects.

Future studies should combine microbiome sequencing, metabolomics, lipidomics, redox proteomics, mitochondrial phenotyping, and ferroptosis-sensitive lipid peroxide profiling. Trials should stratify patients by baseline microbial ecology, diet, renal function, medication exposure, inflammatory state, and redox biomarkers. Rather than testing antioxidants as isolated supplements, the next generation of interventions may combine dietary patterns, microbiota-targeted therapy, mitochondrial support, and ferroptosis modulation. Such designs are more complicated, but they better match the biology of the gut microbiota–mitochondria ferroptosis framework.

For clinical translation, evidence should be interpreted in tiers rather than as a binary positive-or-negative result. The first tier is exposure evidence, showing that a dietary component, probiotic, postbiotic, or redox-active compound reaches the gut or target tissue in a measurable form. The second tier is target engagement, such as altered microbial metabolite profiles, improved barrier markers, restored mitochondrial respiration, or reduced oxidized phospholipid burden. The third tier is surrogate efficacy, including improved endothelial function, hepatic steatosis markers, renal injury markers, or cardiac functional indices. The fourth tier is patient-centered benefit, such as reduced cardiovascular events, kidney decline, hospitalization, or disease progression. Many current antioxidant and microbiota studies reach only the first two tiers, which helps explain why mechanistic promise has not consistently translated into clinical outcomes.

Several experimental design principles follow from this framework. First, studies should distinguish taxonomic microbiome changes from functional metabolite changes; the latter are more directly connected to mitochondrial and ferroptosis biology. Second, mitochondrial assays should include respiration, membrane potential, dynamics, mitophagy, and mtDNA release where possible, not only total ROS. Third, ferroptosis should be inferred from convergent evidence: lipid peroxide species, labile iron, GPX4/SLC7A11 status, ACSL4 or ferritinophagy markers, rescue by ferroptosis inhibitors, and relevant morphology. Fourth, clinical trials should include dietary records, medication data, kidney function, and baseline microbiome features because these variables strongly influence microbial metabolites and antioxidant pharmacokinetics. Fifth, sex, age, menopausal status, and disease stage deserve attention because they may alter bile acid pools, iron handling, mitochondrial reserve, and inflammatory tone. Sixth, trials should predefine target engagement criteria; for example, reduced endotoxemia or TMAO-related exposure, improved mitochondrial respiration or mitophagy markers, reduced oxidized phospholipid burden, or restoration of GSH/GPX4-related lipid peroxide detoxification.

From a translational perspective, the most promising future direction is not a universal antioxidant drug but a stratified intervention logic. Patients with prominent dysbiosis and barrier dysfunction may benefit most from dietary fiber, prebiotics, probiotics, postbiotics, or polyphenol-rich diets. Patients with mitochondrial redox failure may require exercise-mimetic, AMPK/SIRT-linked, CoQ10-related, melatonin, or mitochondria-targeted approaches. Patients with high lipid peroxide and iron signatures may require ferroptosis-sensitive strategies. Patients with persistent inflammatory activation may need combined redox and immune modulation. Multi-omics signatures could eventually assign patients to these intervention classes and monitor whether the gut microbiota–mitochondria ferroptosis framework is being engaged or modified. The key clinical question should shift from “does an antioxidant lower ROS?” to “does this intervention reach the relevant compartment, engage a defined checkpoint, and improve patient-centered outcomes?”.

**Table 6:** Translational challenges and future directions for targeting the gut microbiota–mitochondria ferroptosis framework.

Challenge	Current Limitation	Why It Matters	Future Direction
Causality of gut microbiota	Many studies remain associative and do not establish that dysbiosis initiates ferroptosis-sensitive injury	Correlation may reflect diet, medication, kidney function, host genetics, or disease severity	FMT, germ-free animals, antibiotic rescue designs, metabolite add-back experiments, Mendelian randomization
Specificity of oxidative stress markers	ROS, MDA, and GSH are often nonspecific	General oxidative stress is difficult to distinguish from ferroptosis	Lipidomics, C11-BODIPY, 4-HNE, GPX4, SLC7A11, ACSL4, labile iron assays
Bioavailability of antioxidants	Many polyphenols have low absorption and extensive microbial metabolism	Parent compounds may not represent the active exposure	Nano-delivery, metabolite-focused pharmacology, gut-derived bioactive form analysis, pharmacokinetic sampling
Redox paradox	Excess antioxidant exposure may impair physiological redox signaling	Nonselective ROS suppression may blunt adaptation or reduce efficacy	Dose–response studies and compartment-specific redox targeting
Disease heterogeneity	Cardiometabolic disorders involve multiple organs and disease stages	One intervention is unlikely to fit all phenotypes	Precision nutrition, microbiome stratification, organ-specific redox biomarkers
Lack of clinical validation	Most mechanistic evidence comes from cell and animal models	Human relevance and therapeutic windows remain uncertain	Trials integrating microbiome, metabolomics, lipidomics, redox, ferroptosis biomarkers, and clinical endpoints

## 10. Conclusions

The gut microbiota–mitochondria ferroptosis framework offers a coherent but still incompletely validated redox model for cardiometabolic diseases. Gut dysbiosis and microbial metabolite imbalance may contribute to systemic inflammatory and metabolic pressure; mitochondria may translate those signals into compartment-specific reactive-species production and redox dysfunction; ferroptosis-sensitive pathways may convert lipid peroxide stress into regulated cell injury or death; and inflammatory pathways may propagate tissue injury across the heart, vasculature, liver, adipose tissue, and kidney. However, current evidence often supports local links rather than a complete dysbiosis–metabolite–mitochondria ferroptosis–organ dysfunction chain in the same human study. Because this article is a narrative review, the framework should be regarded as a conceptual model for organizing evidence and generating testable hypotheses rather than as a systematically proven human disease pathway. Redox-targeted interventions are most compelling when they act on this network rather than on ROS alone. Polyphenols, flavonoids, probiotics, prebiotics, postbiotics, melatonin, CoQ10, mitochondria-targeted antioxidants, and precision nutrition may be repositioned as mechanism-defined redox modulators, but their clinical value will depend on better biomarkers, causal microbiome evidence, pharmacokinetic and metabolite exposure data, dose optimization, patient stratification, and human studies that explicitly test whether mechanisms observed in cells and animals operate in clinically relevant cardiometabolic settings.

## Figures and Tables

**Table 1:** Evidence-strength matrix for interpreting local-chain and complete-framework evidence.

Level	Minimum Evidence	Interpretation	Preferred Wording
Level 1	Microbiota, microbial metabolite, redox, or ferroptosis marker associated with cardiometabolic disease	Association or biological plausibility; does not establish directionality or mechanism	Associated with; linked to; candidate marker
Level 2	Microbiota or metabolite manipulation accompanied by mitochondrial or redox changes	Local gut–mitochondrial/redox link; causality depends on model and controls	May modulate; supports a local-chain mechanism
Level 3	Microbiota or metabolite manipulation accompanied by ferroptosis-related changes, with pharmacologic or genetic rescue where possible	Stronger local-chain evidence linking gut-derived exposure to lipid peroxide or ferroptosis-sensitive injury	Ferroptosis-associated or ferroptosis-sensitive injury; mechanistically supported in the model
Level 4	Same experiment tests gut or metabolite exposure, mitochondrial function, ferroptosis criteria, and functional or clinical outcome	Most complete framework evidence; still requires human validation when based on animals or cells	Supports the proposed framework in this model; not necessarily proven in humans

## Data Availability

No new data were generated or analyzed in this study. The literature search strategy, selection rationale, and supplementary evidence tables are described in the manuscript and Supplementary Materials. Additional working bibliography files are available from the corresponding author upon reasonable request.

## Supplementary Materials

The following supporting information can be downloaded at: https://www.mdpi.com/article/10.3390/antiox15070803/s1, Table S1, Evidence map of representative primary and secondary studies relevant to the gut-microbiota-mitochondria-ferroptosis framework [10,11,20,21,24,29,30,44,47,59,66,75,76,79,93,94,95]; Table S2, Candidate biomarkers classified by clinical feasibility; Table S3, PubMed search strategy and screening summary.

## Abbreviations

The following abbreviations are used in this manuscript:

**ACSL4**	acyl-CoA synthetase long-chain family member 4
**AhR**	aryl hydrocarbon receptor
**AMPK**	AMP-activated protein kinase
**CKD**	chronic kidney disease
**CoQ10**	coenzyme Q10
**cGAS-STING**	cyclic GMP-AMP synthase-stimulator of interferon genes
**DHODH**	dihydroorotate dehydrogenase
**ER**	endoplasmic reticulum
**FMT**	fecal microbiota transplantation
**FSP1**	ferroptosis suppressor protein 1
**FXR**	farnesoid X receptor
**GPX4**	glutathione peroxidase 4
**GSH**	glutathione
**IL**	interleukin
**IL-1β**	interleukin-1β
**LPS**	lipopolysaccharide
**MASLD**	metabolic dysfunction-associated steatotic liver disease
**MDA**	malondialdehyde
**mtDNA**	mitochondrial DNA
**mtROS**	mitochondrial reactive oxygen species
**NAFLD**	nonalcoholic fatty liver disease
**NF-κB**	nuclear factor-κB
**NLRP3**	NOD-like receptor family pyrin domain containing 3
**Nrf2**	nuclear factor erythroid 2-related factor 2
**OXPHOS**	oxidative phosphorylation
**ROS**	reactive oxygen species
**SCFAs**	short-chain fatty acids
**SLC7A11**	solute carrier family 7 member 11
**system x_c_^−^**	cystine/glutamate antiporter system
**TGR5**	Takeda G protein-coupled receptor 5
**TMAO**	trimethylamine N-oxide
**TLR4**	Toll-like receptor 4
**TNF-α**	tumor necrosis factor-α
