# Supplementary material for: Linking Gut Microbiota, Mitochondrial Redox Dysfunction, and Ferroptosis in Cardiometabolic Diseases: A Narrative Review of Mechanistic Evidence and Redox-Targeted Interventions
Source: Antioxidants (Basel). 2026 Jun 27;15(7):803. doi: 10.3390/antiox15070803 (PMC13405568; doi:10.3390/antiox15070803)
Supplement: Supplementary file 1 [file antioxidants-15-00803-s001.zip › antioxidants-4366516-supplementary.pdf]

Supplementary Tables

Table S1: Supplementary Table S1. Evidence map of representative primary and secondary studies relevant to the gut-microbiota-mitochondria-ferroptosis framework. NR, not reported in the screened PubMed export or citation record used for this narrative evidence map.

Study / year	Evidence category	Model or population	Sample, sex, dose, duration	Disease context	Gut microbiota or metabolite readout	Mitochondrial or redox readout	Ferroptosis readout or rescue	Complete chain tested?	Evidence level	PMID / DOI	Main limitation
Luo et al., 2024	Primary cell mechanism	Macrophage foam-cell model	Cell model; TMAO dose and duration NR	Atherosclerosis	TMAO used as microbial-host co-metabolite exposure; no microbiome sequencing	Oxidative stress and lipid accumulation markers	Not designed as definitive ferroptosis study	No	1–2	PMID 37932654; DOI 10.1007/s13105-023-00984-y [1]	Cell exposure cannot establish human causality; TMAO is diet- and kidney-function-sensitive
Zhao et al., 2025	Primary animal multi-omics	Experimental atherosclerosis-related comorbidity	Animal model; sex, dose, and duration NR	Atherosclerosis	Metagenomic remodeling	Lipidomic and proteomic redox-metabolic changes	Ferroptosis-related lipid and pathway signals	Partial	3	PMID 41014671; DOI 10.1016/j.phymed.2025.157301 [2]	Compound exposure and comorbidity model require human validation
Ma et al., 2025	Primary animal multi-omics	Experimental steatotic liver disease	Animal model; polysaccharide dose and duration NR	MASLD/MASH-related injury	Microbiota and metabolite remodeling	Reduced oxidative metabolic stress	Gut microbiota-metabolite-ferroptosis pathway markers	Partial	3	PMID 40570246; DOI 10.1021/acs.jafc.5c05877 [3]	Active fractions, dose translation, and human exposure remain uncertain
Liu et al., 2025	Primary animal causal manipulation	Metabolism-associated fatty liver disease model	Animal model; ginsenoside Rd dose and duration NR	MASLD/MAFLD terminology in source	Metagenomics, antibiotic cocktail, and fecal-transfer related evidence in abstract	Improved mitochondrial damage and redox stress	Lipid peroxidation and ferroptosis pathway markers	Partial in animals	3–4	PMID 40495179; DOI 10.1186/s13020-025-01121-1 [4]	Strong preclinical design but not a human complete-chain study
Gu et al., 2025	Primary animal pharmacology	MASLD experimental model	Animal model; botanical dose and duration NR	MASLD	Gut microbiota and linoleic-acid metabolism	Oxidative stress readouts	9(S)-HpODE and GPX4-mediated ferroptosis readouts	Partial	3	PMID 40334428; DOI 10.1016/j.phymed.2025.156806 [5]	Botanical mixture and metabolite specificity need independent validation
Zhang et al., 2026	Primary animal intervention	Diabetic liver injury model	Animal model; propyl gallate dose and duration NR	Diabetic liver injury	Gut-liver axis modulation	Hepatic redox injury markers	SLC7A11/GPX4-mediated hepatic ferroptosis markers	Partial	3	PMID 41876017; DOI 10.1016/j.bcp.2026.117926 [6]	Diabetic liver model does not establish broader cardiometabolic outcomes
Wu et al., 2026	Primary animal intervention	Hepatic fibrosis model	Animal model; polysaccharide dose and duration NR	Liver fibrosis/MASLD-related injury	Gut microbiota dependence reported in title and abstract	Oxidative and inflammatory injury markers	Suppression of ferroptosis-associated injury	Partial	3	PMID 41966031; DOI 10.1016/j.phymed.2026.158123 [7]	Fibrosis model and polysaccharide composition may not translate directly to humans
Zhao et al., 2026	Primary animal intervention	Liver fibrosis model	Animal model; fructan dose and duration NR	Liver fibrosis/MASLD-related injury	Microbiota-dependent effect	Redox injury and fibrosis markers	Ferroptosis inhibition markers	Partial	3	PMID 42002330; DOI 10.1016/j.carbpol.2026.125243 [8]	Preclinical and product-specific; complete human chain not tested
Tong et al., 2023	Primary animal inhibitor study	Mouse metabolic liver disease model	Mouse model; liproxstatin-1 dose and duration NR	MASLD	Not gut-primary	Reduced lipid peroxidation and inflammatory injury	ACSL4/ALOX15, 4-HNE, MDA, and related markers; pharmacologic inhibition	No	Supportive	PMID 36323829; DOI 10.1038/s41401-022-01010-5 [9]	Supports ferroptosis relevance but does not test microbiota causality
Wu et al., 2024	Primary animal intervention	Experimental diabetic cardiomyopathy	Animal model; paeoniflorin dose and duration NR	Diabetic cardiomyopathy	Gut microbiota and metabolite changes	Cardiac redox injury markers	Ferroptosis-resistance markers	Partial	3	PMID 38223927; DOI 10.1152/ajpcell.00565.2023 [10]	Requires human cardiac-function and biomarker validation
Wang et al., 2022	Primary animal/cell mechanism	Diabetic nephropathy model	Animal/cell model; schisandrin A dose and duration NR	Diabetic kidney disease	Not gut-primary	Mitochondrial damage and AdipoR1-related signaling	Ferroptosis and NLRP3 inflammasome markers	No	Supportive	PMID 35996380; DOI 10.1155/2022/5411462 [11]	Strong local mitochondrial-ferroptosis evidence but not a gut-chain test
Zhao et al., 2026	Primary animal gut-kidney intervention	Diabetic kidney disease model	Animal model; nobiletin dose and duration NR	Diabetic kidney disease	Gut-kidney axis and microbiota-related readouts	Oxidative injury and renal remodeling markers	Ferroptosis and epithelial-mesenchymal transition markers	Partial	3	PMID 41582083; DOI 10.1142/S0192415X26500114 [12]	Human relevance, active metabolite exposure, and renal confounding remain unresolved
Liang et al., 2025	Primary animal probiotic study	Myocardial ischemia-reperfusion model	Animal model; Lactobacillus strain, dose, and duration NR	Myocardial injury	Probiotic modulation of gut microbial ecology	Oxidative stress and inflammatory markers	Ferroptosis-related cardiac protection markers	Partial	3	PMID 40598393; DOI 10.1186/s12916-025-04203-x [13]	Acute injury model; strain-specific clinical translation uncertain
Zhao et al., 2024	Primary animal nutritional intervention	Iron-overload model	Animal model; L-citrulline dose and duration NR	Iron stress with metabolic relevance	Improved microbiota composition	AMPK-linked mitochondrial quality-control markers	Iron metabolism and ferroptosis-tendency readouts	Partial	3	PMID 38425278; cited in main text [14]	Iron-overload model is not a classic cardiometabolic disease model
Costa et al., 2026	Secondary translational review	Diabetic kidney disease literature	Not applicable	Diabetic kidney disease	Probiotic modulation summarized	Oxidative stress and inflammatory endpoints summarized	Ferroptosis not consistently measured	No	Secondary	PMID 41525345; DOI 10.1590/2175-8239-JBN-2025-0143en [15]	Review-level evidence; few trials include ferroptosis endpoints

Study / year	Evidence category	Model or population	Sample, sex, dose, duration	Disease context	Gut microbiota or metabolite readout	Mitochondrial or redox readout	Ferroptosis readout or rescue	Complete chain tested?	Evidence level	PMID / DOI	Main limitation
Xu et al., 2024	Secondary RCT meta-analysis	Randomized trials in heart failure	Trial sample, dose, and duration varied by included study	Heart failure	Not gut-primary	CoQ10 redox biology and cardiovascular outcomes inferred	Ferroptosis not directly measured	No	Clinical redox	PMID 39462324; DOI 10.1186/s12872-024-04232-z [16]	Heterogeneous formulations and endpoints; no microbiota-ferroptosis readouts
Daei et al., 2024	Secondary RCT meta-analysis	Randomized trials of endothelial function	Trial sample, dose, and duration varied by included study	Vascular endothelial function	Not gut-primary	Endothelial-function and oxidative-stress surrogate outcomes	Ferroptosis not directly measured	No	Clinical redox	PMID 38630421; DOI 10.1007/s40292-024-00630-8 [17]	Surrogate outcomes predominate; no direct gut or ferroptosis target engagement

Table S2: Supplementary Table S2. Candidate biomarkers classified by clinical feasibility for evaluating the gut-microbiota-mitochondria-ferroptosis framework.

Feasibility class	Biomarker group	Detection methods	Interpretation	Key limitation
Clinical feasible	TMAO, trimethylamine-related metabolites, SCFAs, bile acids, indoles, phenolic acids, uremic solutes	Targeted LC-MS/MS, GC-MS, clinical metabolomics	Functional microbiota-host metabolic output; useful for exposure and stratification	Levels reflect diet, kidney function, liver metabolism, medications, and sampling time
Clinical feasible	LPS, LBP, soluble CD14, intestinal fatty-acid-binding protein, selected permeability assays	ELISA, immunoassay, permeability testing	Barrier dysfunction and microbial product translocation	Circulating assays are indirect and not fully standardized across laboratories
Clinical feasible	hsCRP, IL-6, TNF-α, IL-1β, routine liver enzymes, eGFR, albuminuria, lipid panel, HbA1c	Clinical chemistry, ELISA, multiplex panels	Disease severity, inflammatory tone, and confounding clinical context	Nonspecific; cannot distinguish upstream gut effects from downstream tissue injury
Clinical feasible	Circulating oxidative-stress markers such as MDA, 4-HNE adducts, F2-isoprostanes, GSH/GSSG, antioxidant enzyme activity	ELISA, colorimetric assays, LC-MS/MS	Redox burden and lipid peroxide exposure at systemic level	Assay variability and limited tissue specificity; single markers do not diagnose ferroptosis
Translational research	Microbial composition and function, including alpha-diversity, beta-diversity, metagenomic pathways, TMA-producing genes, bile-acid-transforming genes	16S rRNA sequencing, shotgun metagenomics, metatranscriptomics	Microbial community and functional pathway alteration	Taxonomy alone does not establish metabolite output or causality
Translational research	Mitochondrial bioenergetics in accessible cells or biopsies: oxygen consumption, ATP, membrane potential, OXPHOS proteins, $NADH/NAD^+$	Seahorse assay, high-resolution respirometry, JC-1/TMRE, enzymatic assays, Western blot	Respiratory efficiency and energetic reserve	Blood cells may not represent target organs; tissue biopsies limit scalability
Translational research	Mitochondrial quality-control and innate-immune markers: mtDNA release, PINK1/Parkin, LC3-II, p62, DRP1, MFN1/2, SIRT3, AMPK, cGAS-STING	qPCR, Western blot, immunostaining, electron microscopy, targeted proteomics	Mitochondrial damage, mitophagy, dynamics, and inflammatory coupling	Static markers can reflect either adaptive repair or excessive organelle loss
Translational research	Tissue or circulating ferroptosis-associated markers: GPX4, SLC7A11, ACSL4, FSP1, DHODH, ferritin, transferrin receptor, hepcidin, NCOA4	IHC, Western blot, qPCR, ELISA, targeted proteomics	Lipid-peroxide defense, iron handling, and ferroptosis susceptibility	Requires tissue context and multiple markers; isolated changes are insufficient
Experimental only	Direct lipid-peroxidation and labile-iron assays: C11-BODIPY, oxidized phosphatidylethanolamines, Fe^{2+} probes, lipid radical trapping	Flow cytometry, fluorescence microscopy, lipidomics, iron probes	Strong mechanistic evidence for lipid peroxide and iron-dependent injury	Usually cell or tissue based; limited routine clinical feasibility
Experimental only	Rescue or causality tests: ferrostatin-1, liproxstatin-1, iron chelation, GPX4/SLC7A11/ACSL4 genetic manipulation, FMT, germ-free or antibiotic-rescue designs	Pharmacologic rescue, genetic models, microbiota transfer, isotope tracing	Distinguishes association from mechanistic dependence	Often preclinical; intervention specificity and human translation remain uncertain

Table S3: Supplementary Table S3. PubMed search strategy and screening summary used for the narrative evidence map. Searches were conducted during manuscript preparation, with main exports generated on 24 May 2026 and updated through 20 June 2026.

Search file or step	PubMed query or screening action	Exported records	Unique records	Notes
Core framework	((“gut microbiota” OR “gut microbiome” OR “intestinal microbiota” OR “gut dysbiosis” OR “microbial metabolites” OR “gut-derived metabolites” OR TMAO OR “short-chain fatty acids” OR SCFAs OR “bile acids” OR “tryptophan metabolites” OR LPS) AND (“mitochondrial dysfunction” OR “mitochondrial oxidative stress” OR “mitochondrial ROS” OR mtROS OR “mitochondrial redox” OR “redox homeostasis” OR “oxidative stress” OR ROS OR mitophagy) AND (ferroptosis OR “lipid peroxidation” OR “iron overload” OR GPX4 OR SLC7A11 OR ACSL4 OR FSP1) AND (“cardiometabolic disease” OR “cardiovascular disease” OR “metabolic syndrome” OR obesity OR diabetes OR “insulin resistance” OR atherosclerosis OR “diabetic cardiomyopathy” OR MASLD OR NAFLD))	97	97	Broad mechanism search retained for framework construction
Expanded framework	((“gut microbiota” OR “gut microbiome” OR “microbial metabolites” OR TMAO OR SCFAs OR “bile acids” OR LPS) AND (“oxidative stress” OR “redox homeostasis” OR “mitochondrial dysfunction” OR mtROS OR mitophagy) AND (ferroptosis OR “lipid peroxidation” OR GPX4 OR SLC7A11) AND (cardiovascular OR metabolic OR diabetes OR obesity OR atherosclerosis OR MASLD OR NAFLD))	560	559	Used to identify additional mechanistic and disease-context records
Microbial metabolites	((“microbial metabolites” OR “gut-derived metabolites” OR TMAO OR “short-chain fatty acids” OR SCFAs OR “bile acids” OR “tryptophan metabolites” OR LPS) AND (“mitochondrial dysfunction” OR “oxidative stress” OR “redox homeostasis” OR mtROS) AND (ferroptosis OR “lipid peroxidation” OR “iron metabolism” OR GPX4 OR SLC7A11))	517	516	Emphasized metabolite-specific redox and ferroptosis links
Redox-targeted interventions	((“gut microbiota” OR “gut microbiome” OR “microbial metabolites” OR TMAO OR SCFAs OR “bile acids”) AND (“mitochondrial dysfunction” OR “mitochondrial oxidative stress” OR mtROS OR “redox homeostasis”) AND (ferroptosis OR “lipid peroxidation” OR GPX4 OR SLC7A11) AND (antioxidants OR polyphenols OR flavonoids OR resveratrol OR quercetin OR curcumin OR melatonin OR probiotics OR prebiotics OR postbiotics OR nutraceuticals OR “functional foods” OR MitoQ OR “Coenzyme Q10”))	20	20	Used for intervention and pharmacology sections
Diabetes-focused search	((“gut microbiota” OR “gut dysbiosis” OR “microbial metabolites”) AND (“mitochondrial dysfunction” OR “mitochondrial oxidative stress” OR mtROS) AND (ferroptosis OR “lipid peroxidation” OR GPX4 OR ACSL4) AND (diabetes OR “type 2 diabetes” OR “diabetic cardiomyopathy” OR “insulin resistance”))	11	11	Used for diabetes and diabetic cardiomyopathy subsection
MASLD-focused search	((“gut microbiota” OR “gut dysbiosis” OR “microbial metabolites” OR LPS OR “bile acids”) AND (“mitochondrial dysfunction” OR “oxidative stress” OR mtROS) AND (ferroptosis OR “lipid peroxidation” OR GPX4 OR ACSL4) AND (MASLD OR NAFLD OR “fatty liver disease” OR “metabolic dysfunction-associated steatotic liver disease”))	19	19	Included older NAFLD and MAFLD terminology for historical retrieval
Atherosclerosis/vascular search	((“gut microbiota” OR TMAO OR SCFAs OR LPS) AND (“mitochondrial dysfunction” OR “oxidative stress” OR mtROS) AND (ferroptosis OR “lipid peroxidation” OR GPX4 OR SLC7A11) AND (atherosclerosis OR “vascular dysfunction” OR “endothelial dysfunction”))	11	11	Used for vascular disease subsection
Deduplication	Duplicate PubMed records across the broad and focused exports were removed manually by PMID and title matching before narrative screening	1235 exported records across files	696 unique records	The unique count was used as the denominator for screening
Title and abstract screening	Records were retained when they addressed at least one major domain (microbiota/metabolites, mitochondrial redox biology, ferroptosis/lipid peroxidation, cardiometabolic disease, or redox-targeted intervention) and had plausible relevance to the framework	696 screened	150 candidate records	Screening was performed by Y.C., J.Z. and H.G.; disagreements were resolved with M.W.; K.M. and M.L. checked evidence tables
Citation set used in manuscript	Candidate records were cited when they provided disease-relevant primary evidence, mechanistic clarity, human/translational relevance, randomized or genetic evidence, or a contemporary synthesis needed to orient the narrative	150 assessed	112 unique cited references in the main text	Remaining candidates were excluded from citation because they were less disease-relevant, mechanistically duplicative, outside the cardiometabolic scope, review-level statements superseded by primary studies, or lacked a gut, mitochondrial, ferroptosis, or intervention component
Eligibility boundaries	English-language full-text publications were used for the cited evidence set. Searches were not restricted by study design because this was a mechanism-centered narrative review rather than a PRISMA systematic review	Not applicable	Not applicable	No pre-registered protocol, formal risk-of-bias tool, duplicate independent screening, or meta-analysis was performed

References

[1] Z. Luo, X. Yu, C. Wang, H. Zhao, X. Wang, and X. Guan. Trimethylamine n-oxide promotes oxidative stress and lipid accumulation in macrophage foam cells via the nrf2/abca1 pathway. *J Physiol Biochem*, 2024.

[2] Y. Zhao, T. Song, P. Ren, X. Wu, Q. Luo, J. Xie, H. Lai, X. Li, Y. Wen, X. Liao, et al. Integrating metagenomics, lipidomics and proteomics to explore the effect and mechanism of ginsenoside rb1 on atherosclerosis co-depression disease. *Phytomedicine*, 2025.

[3] C. Ma, Y. Bao, S. Hereid, H. Zhang, X. Bai, Q. Bai, L. Zhao, X. Zhang, H. Lian, L. Dai, et al. Mechanistic elucidation of tricholoma mongolicum polysaccharides in treating mafld via regulation of the gut microbiota-metabolite-ferroptosis axis: A multi-omics perspective. *J Agric Food Chem*, 2025.

[4] W. Liu, X. Zhou, L. Xiao, X. Huang, D. Chang, X. Zhong, M. Zeng, Y. Xian, Y. Zheng, W. Huang, et al. The gut microbiota-mediated ferroptosis pathway: a key mechanism of ginsenoside rd against metabolism-associated fatty liver disease. *Chin Med*, 2025.

[5] S. Gu, C. Chen, J. Wang, Y. Wang, L. Zhao, Z. Xiong, H. Zhang, T. Deng, Q. Pan, Y. Zheng, et al. Camellia japonica radix modulates gut microbiota and 9(s)-hpode-mediated ferroptosis to alleviate oxidative stress against masld. *Phytomedicine*, 2025.

[6] Z. Zhang, S. Jiao, H. Wang, Y. Chen, Z. Zhang, Y. Zhang, R. Gao, Y. Xiao, Y. Liu, Y. Zhu, et al. Propyl gallate mitigates diabetic liver injury via suppressing slc7a11/gpx4-mediated hepatic ferroptosis and modulating gut-liver axis. *Biochem Pharmacol*, 2026.

[7] J. Wu, Z. Yang, L. Chen, Q. Xu, Y. Zhang, Y. Yang, M. Beloved, S. Zhan, W. Cao, Z. Li, et al. A polysaccharide from pueraria lobata ameliorates hepatic fibrosis via gut microbiota-dependent suppression of ferroptosis. *Phytomedicine*, 2026.

[8] Y. Zhao, M. Qiao, C. Ma, Q. Hou, J. Hu, and J. Yang. A fructan-type polysaccharide from lycium ruthenicum attenuates liver fibrosis via microbiota-dependent ferroptosis inhibition. *Carbohydr Polym*, 2026.

[9] J. Tong, X. T. Lan, Z. Zhang, Y. Liu, D. Y. Sun, X. J. Wang, S. X. Ou-Yang, C. L. Zhuang, F. M. Shen, P. Wang, et al. Ferroptosis inhibitor liproxstatin-1 alleviates metabolic dysfunction-associated fatty liver disease in mice: potential involvement of panoptosis. *Acta Pharmacol Sin*, 2023.

[10] H. Wu, P. Zhang, J. Zhou, S. Hu, J. Hao, Z. Zhong, H. Yu, J. Yang, J. Chi, and H. Guo. Paeoniflorin confers ferroptosis resistance by regulating the gut microbiota and its metabolites in diabetic cardiomyopathy. *Am J Physiol Cell Physiol*, 2024.

[11] X. Wang, Q. Li, B. Sui, M. Xu, Z. Pu, and T. Qiu. Schisandrin a from schisandra chinensis attenuates ferroptosis and nlrp3 inflammasome-mediated pyroptosis in diabetic nephropathy through mitochondrial damage by adipor1 ubiquitination. *Oxid Med Cell Longev*, 2022.

[12] T. Zhao, C. Zhao, Q. Xiang, X. Zhang, K. F. Hong, P. Liu, Z. Sun, Y. Liu, R. Huang, Y. Li, et al. Nobiletin ameliorated the development of diabetic kidney disease via modulating ferroptosis and epithelial-mesenchymal transition involving gut-kidney axis. *Am J Chin Med*, 2026.

[13] Y. Liang, L. Zhao, X. Zhang, S. Liu, P. Lu, J. Wang, L. Chen, Y. Liu, and J. Gao. Lactobacillus ameliorates myocardial ischemia reperfusion injury by attenuating apoptosis, inflammation, oxidative stress, and ferroptosis. *BMC Med*, 2025.

[14] D. Zhao, Y. Gao, Y. Chen, Y. Zhang, Y. Deng, S. Niu, and H. Dai. L-citrulline ameliorates iron metabolism and mitochondrial quality control via activating ampk pathway in intestine and improves microbiota in mice with iron overload. *Mol Nutr Food Res*, 2024.

[15] V. C. S. Costa, M. M. Pinheiro, G. T. Cabreira, I. B. Bustelli, J. F. Santos, S. Ventura, L. S. C. Santos, M. F. F. Vattimo, and E. O. Silva. The role of probiotics in modulating the gut microbiota as a potential inhibitor of diabetic kidney disease progression. *J Bras Nefrol*, 2026.

[16] J. Xu, L. Xiang, X. Yin, H. Song, C. Chen, B. Yang, H. Ye, and Z. Gu. Efficacy and safety of coenzyme q10 in heart failure: a meta-analysis of randomized controlled trials. *BMC Cardiovasc Disord*, 2024.

[17] S. Daei, A. Ildarabadi, S. Goodarzi, and M. Mohamadi-Sartang. Effect of coenzyme q10 supplementation on vascular endothelial function: A systematic review and meta-analysis of randomized controlled trials. *High Blood Press Cardiovasc Prev*, 2024.
